# Experimental and Theoretical Investigation of Ion Pairing in Gold(III) Catalysts

**DOI:** 10.1021/acs.organomet.3c00293

**Published:** 2023-09-30

**Authors:** Jacopo Segato, Eleonora Aneggi, Walter Baratta, Filippo Campagnolo, Leonardo Belpassi, Paola Belanzoni, Daniele Zuccaccia

**Affiliations:** † Dipartimento di Scienze Agroalimentari, Ambientali e Animali, Sezione di Chimica, Università di Udine, Via Cotonificio 108, I-33100 Udine, Italy; ‡ Istituto di Scienze e Tecnologie Chimiche (SCITEC), Consiglio Nazionale delle Ricerche c/o Dipartimento di Chimica, Biologia e Biotecnologie, Università degli Studi di Perugia, Via Elce di Sotto 8, 06123 Perugia, Italy; § Dipartimento di Chimica, Biologia e Biotecnologie, Università degli Studi di Perugia, 06123 Perugia, Italy

## Abstract

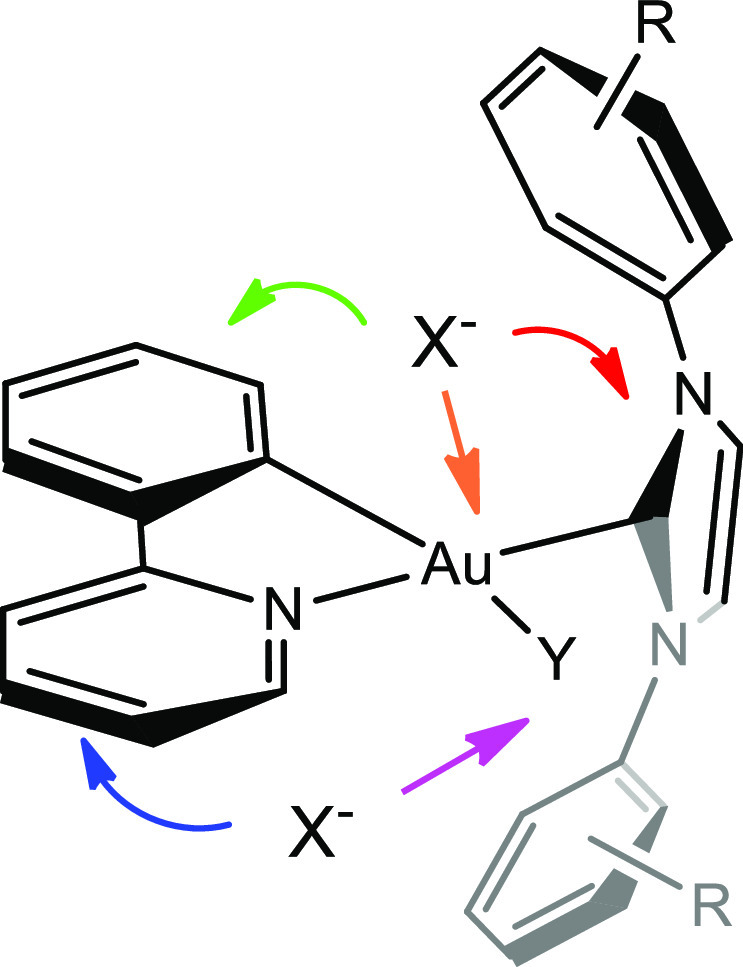

The ion pairing structure of the possible species present in solution during the gold(III)-catalyzed hydration of alkynes: [(ppy)Au(NHC)Y]X_2_ and [(ppy)Au(NHC)X]X [ppy = 2-phenylpyridine, NHC = NHC^iPr^ = 1,3-bis(2,6-di-isopropylphenyl)-imidazol-2-ylidene; NHC = NHC^mes^ = 1,3-bis(2,4,6-trimethylphenyl)-imidazol-2-ylidene X = Cl^–^, BF_4_^–^, OTf^–^; Y = H_2_O and 3-hexyne] are determined. The nuclear overhauser effect nuclear magnetic resonance (NMR) experimental measurements integrated with a theoretical description of the system (full optimization of different ion pairs and calculation of the Coulomb potential surface) indicate that the preferential position of the counterion is tunable through the choice of the ancillary ligands (NHC^iPr^, NHC^mes^, ppy, and Y) in [(ppy)Au(NHC)(3-hexyne)]X_2_ activated complexes that undergo nucleophilic attack. The counterion can approach near NHC, pyridine ring of ppy, and gold atom. From these positions, the anion can act as a template, holding water in the right position for the outer-sphere attack, as observed in gold(I) catalysts.

## Introduction

While gold(I) catalysis is a vitally important area of research with many reported examples,^1^ gold(III)-catalyzed reactions are still in their infancy.^2^ In addition, the mechanistic proposals for reactions involving Au(III) catalysts^3^ most often lack reliability^4^ if compared to gold(I) catalysts, where the role of ligand, counterion, substrate, nucleophile, solvent, and additives are well established.^5^

Over the past few years, even some of us have been engaged in the rationalization from both experimental and theoretical point of view of important features of gold(I) catalysis.^6^ Kinetic experiments, together with multinuclear and multidimensional NMR measurements and density functional theory (DFT) calculations, permit us to study, understand, and rationalize the importance of both counterion^7^ (in terms of gold-counterion affinity and hydrogen bond basicity) and ligand^8^ (in terms of donation and π-backdonation properties versus gold) in the catalytic cycle (preequilibrium step, nucleophilic attack, protodeauration, gold deactivation). Moreover, we have pointed out the crucial role of solvent and noncovalent interactions from both experimental and theoretical point of view.^9^ These results allowed us to develop, for the first time, a green strategy for hydration of alkynes promoted by gold(I) species in both neat conditions^10^ and in green solvents.^11^

The foundations underlying this recent rationalization of gold chemistry, however, are to be found in the preliminary determination of the ion pairing structure of [L-Au(I)-S]^+^X^–^ (L = carbenes and phosphanes, S = unsaturated hydrocarbons, and X^–^ = weakly coordinating counterion) systems that are the most important intermediates formed during gold-catalyzed nucleophilic additions to an unsaturated substrate. The anion in order to influence the kinetics of the reaction must be in the correct position, at least at rate determining step (RDS) of the reaction.^12^ Since 2009,^13^ several interionic characterization^14^ of [L-Au(I)-S]^+^X^–^ species have been made by some of us taking advantage of nuclear overhauser effect (NOE) NMR experiments and DFT calculations of potential energy surfaces (PES) and Coulomb potential of the ions. These powerful experimental and theoretical methods are used by us for understanding the relative anion–cation orientation determined by the nature of the ancillary ligand (L), substrate (S), and counterion (X^–^). This fine-tuning of the interionic structure has paved the way to larger control over the properties and activity of these catalysts.^15^ Similar studies on the ion pairing in gold(III) chemistry are not available yet.

Recently, the mechanism of the hydration of 3-hexyne catalyzed by [(ppy)Au^(III)^(NHC^iPr^)Cl]Cl [ppy = 2-phenylpyridine, NHC^iPr^ = 1,3-bis(2,6-di-isopropylphenyl)-imidazol-2-ylidene] in GVL as solvent has been investigated by some of us both experimentally (NMR) and computationally (DFT),^16^ demonstrating that the pre-equilibrium step is effectively the RDS. As a matter of fact, water or counterion substitution by 3-hexyne in the first co-ordination sphere of Au(III) is crucial for the whole process.^17^

In this work, we focus on the ion pairing structure determined by NOE NMR spectroscopy of the possible species present in solution during the catalysis: [(ppy)Au(NHC)Y]X_2_ and [(ppy)Au(NHC)X]X [ppy = 2-phenylpyridine, NHC = NHC^iPr^ = 1,3-bis(2,6-di-isopropylphenyl)-imidazol-2-ylidene; NHC = NHC^mes^ = 1,3-bis(2,4,6-trimethylphenyl)-imidazol-2-ylidene X = Cl^–^, BF_4_^–^, OTf^–^; Y = H_2_O and 3-hexyne]. The NOE NMR experimental data were also integrated with a theoretical description of the system, through full optimization of different ion pairs and calculation of the Coulomb potential surface (see Computational Details). This integrated NMR/DFT approach has already been demonstrated to be effective and useful^18^ to give an in-depth comprehension and rationalization of experimental data. For the first time, the determination of the relative anion–cation orientation(s) in gold(III) complexes is reported.

## Results and Discussion

### Synthesis, Intramolecular, and Interionic Characterization by NMR Experiments of Gold(III) Complexes

**Figure 1:**
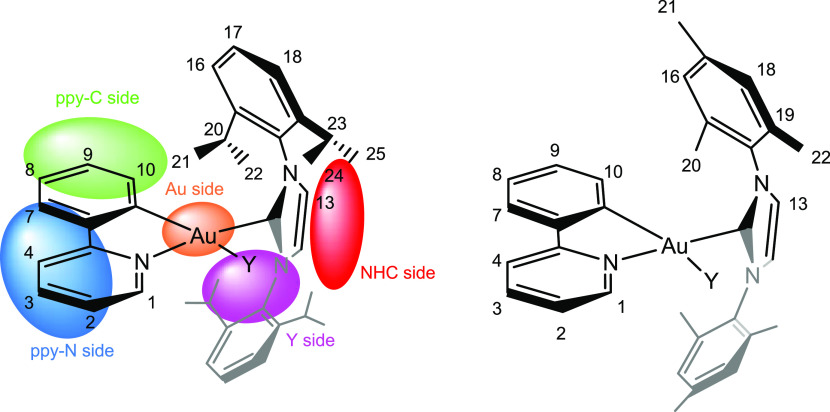
Structure of the cation, numbering of the most relevant resonances of complex of the type [(ppy)Au(NHC)Y]X_2_ and the most important regions utilized for the assessment of the ion pairing structure: NHC side (red), ppy-C side (green), ppy-N side (blue), Y side (magenta), and Au (orange).

The first complex that can be formed during the pre-equilibrium step of the catalytic cycle is [(ppy)Au(NHC^iPr^)Cl]OTf in which noncoordinating chloride counterion that has been replaced by triflate is replaced by OTf with the help of AgOTf. This complex was synthesized according to the literature.^16,17^ The characterization of complex by mono and bidimensional ^1^H, ^13^C, and ^19^F NMR experiments confirms the structure of the cation (relative orientation of ligands around the metal center) previously observed for [(ppy)Au(NHC^iPr^)Cl]^+16,17^ and reported in Figure 1 together with the numbering of the proton atoms. The most important features of the structure of the cation are that NHC and ppy ligands are coordinated to the metal center with NHC trans to the N atom of ppy and that those ligands are perpendicular to each other, generating four different signals for the methyl groups of the isopropyl fragments of NHC (Figure 1). The same structure of the cation is observed for [(ppy)Au(NHC^iPr^)Cl]BF_4_ and [(ppy)Au(NHC^iPr^)H_2_O](BF_4_)_2_.^16,17^ In addition, the same configuration is observed for [(ppy)Au(NHC^iCy^)Cl]BF_4_ [NHC^iCy^ = 1,3-dicyclohexyl-imidazol-2-ylidene] in solid state and predicted by DFT calculations.^16,17^

The complete assignment of all ^1^H resonances and subsequently the structure of the cation is mandatory in order to obtain information on relative anion–cation position (ion pairing structure, Figure 1) by the ^19^F, ^1^H-HOESY NMR spectrum technique. Given the “complicated and unsymmetrical” intramolecular structure of the cation, since several inequivalent “reporter” nuclei may assess the relative anion–cation orientation, different noteworthy regions can be identified where the anion can locate which are near the imidazolium ring (Figure 1, NHC side) and on the side of the pyridine of phenylpyridine ligand (Figure 1, ppy-N side), close to the ligand Y (Cl^–^, OTf^–^, H_2_O) (Figure 1, Y side), close to the C–Au bond of phenylpyridine ligand (Figure 1, ppy-C side), and close to gold (Figure 1, Au-side)

**Figure 2:**
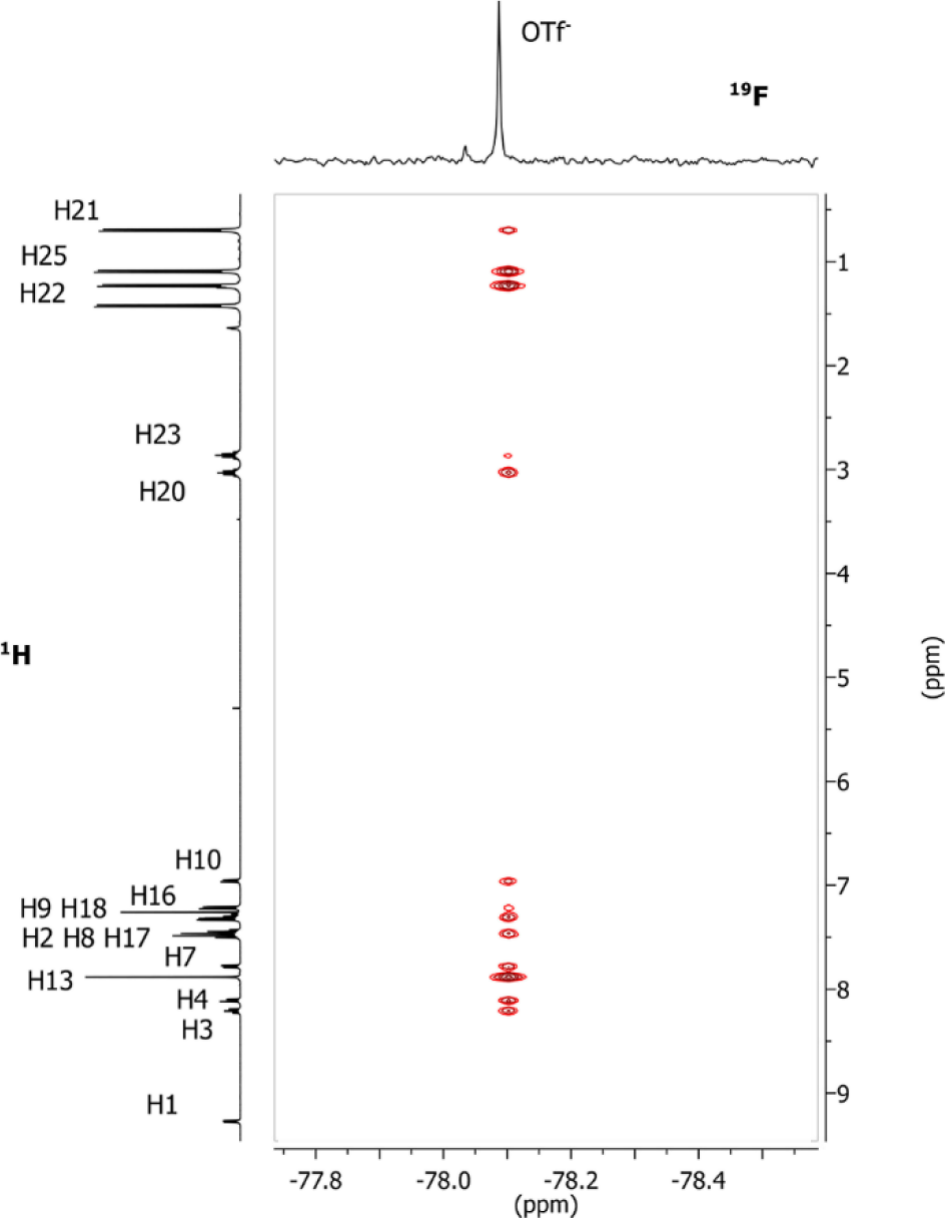
^19^F,^1^H-HOESY NMR spectrum (376.65 MHz, 297 K, CD_2_Cl_2_) of the complex [(ppy)Au(NHC^iPr^)Cl]OTf.

In order to understand the position of the counterion around the cationic metal fragment, we recorded a ^19^F, ^1^H-HOESY NMR spectrum (Figure 2). By analyzing the ^19^F, ^1^H-HOESY NMR spectrum, it is evident that the anion interacts with all of the protons of the phenylpyridine ligand except H1 and with all of the protons of the NHC ligand except H24. Both protons are located near Cl. The most intense signals are with the protons of the imidazole (H13) and with the methyl protons (H22 and H25) that point to the imidazole ring. Medium-strength contacts were observed with the protons of the phenylpyridine (H3, H4, H7, and H8) which overlook the opposite site of gold. Weak contacts are present with the aromatic protons (H16, H17, and H18) of the NHC and with the isopropyl hydrogens H20 and H23. Weak signals are also present with the protons of the phenylpyridine H2, H9, and H10.

By normalizing all the NOE interionic contacts for the number of magnetically equivalent nuclei (Table 1),^19^ the H13/F peak is the most intense and normalized to one (entry 11) followed by H25 and H23 (entries 2 and 3). Medium-lower values around 0.29–0.21 of normalized NOE intensities are found for H20, H10, H7, H7, H4, and H3 (entries 5, 6, 10, 12, and 13, respectively)

**Table 1:** Normalized (*I*/*f*) NOE Intensities for [(ppy)Au(NHC^iPr^)Cl]OTf, Determined Arbitrarily by Fixing at 1 the Normalized Intensity of the NOE between the Anion and H13^a^

entry	^1^H	(*I*/*f*) NOE
1	21	0.07
2	25	0.37
3	22	0.52
4	23	0.03
5	20	0.22
6	10	0.21
7	16	0.05
8	9–18	0.14
9	2–8–17	0.12
10	7	0.27
11	13	1.00
12	4	0.29
13	3	0.28

a The scaling factor *f* is equal to (*n*H × *n*F)/(*n*H + *n*F), where *n* is the number of magnetically equivalent nuclei (H or F).

This pattern of NOE clearly indicates that the most favorable position of the counterion is near the imidazole ring (Figure 1, NHC side) even if other positions near the backbone of phenylpyridine (Figure 1, ppy-N side), near gold (Figure 1, Au side), and near phenyl of ppy (Figure 1, ppy-C side) are accessible by the counterion. Finally, the anion is located far away from the chlorine (Figure 1, Y–Au side) that we assume is staying in the inner sphere (while the triflate is always in the second sphere, as suggested by the ^19^F NMR spectrum).

The same ion pairing structure was observed for the complex [(ppy)Au(NHC^iPr^)Cl]BF_4_ synthesized in an NMR tube and fully characterized (see SI). The preferred position of the counterion is near the NHC imidazole ring (NHC-side, Figure 1).

The second important complex formed during the pre-equilibrium step of the catalytic cycle is [(ppy)Au(NHC^iPr^)OTf]OTf in which both chlorines are replaced by OTf^–^ with the help of AgOTf.

**Figure 3:**
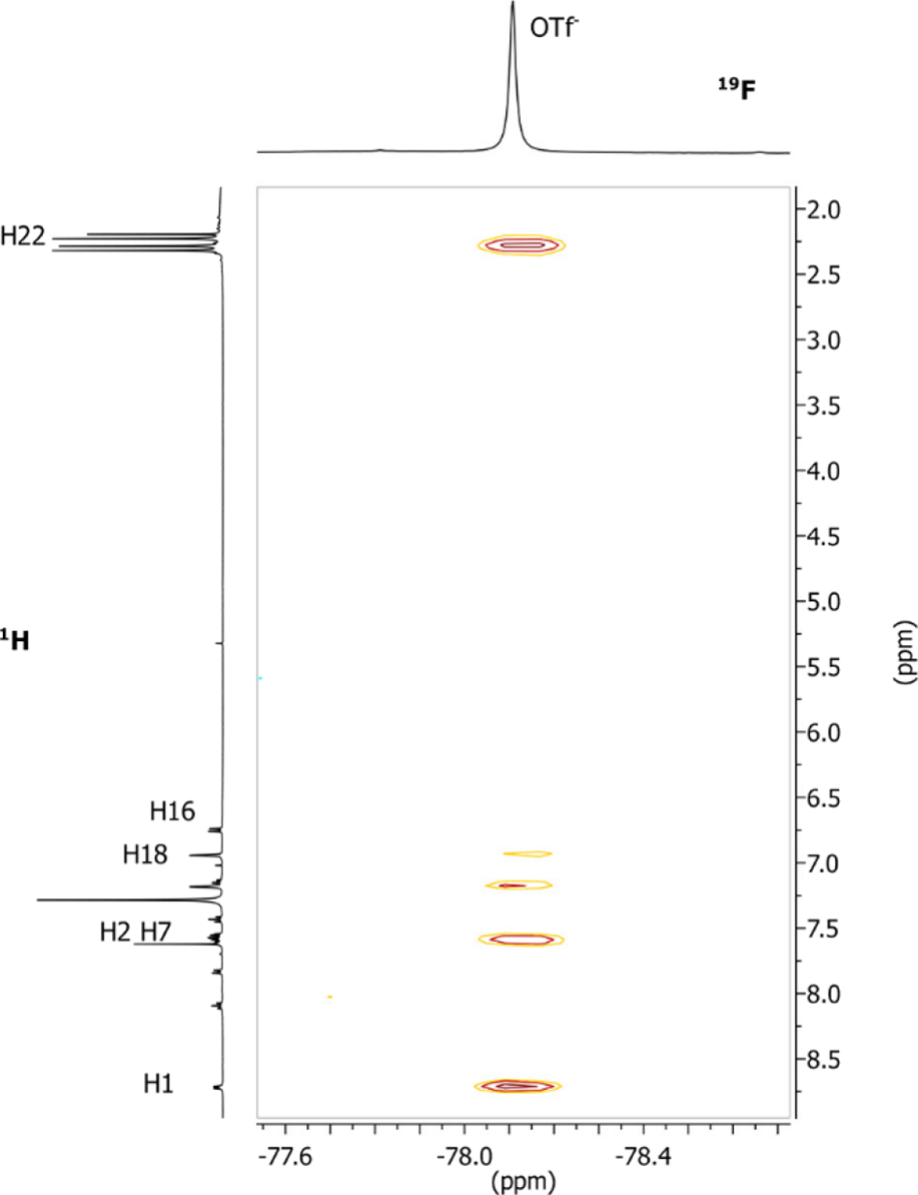
^19^F, ^1^H-HOESY NMR spectrum (376.65 MHz, 297 K, CD_2_Cl_2_) of the complex [(ppy)Au (NHC^mes^)OTf]OTf.

Unfortunately, any attempts to synthesize, isolate, and characterize [(ppy)Au(NHC^iPr^)OTf]OTf have failed. As previously observed^16,17^ [(ppy)Au(NHC^iPr^)OTf]OTf, generated in situ in an NMR tube, is mixed with [(ppy)Au(NHC^iPr^)Cl]OTf and any attempt of purification did not lead to the desired results. Therefore, we did not analyze the ion pairing structure because the ^19^F, ^1^H-HOESY NMR spectrum could not be analyzed due to its difficult evaluation. Similarly, also for [(ppy)Au(NHC^iPr^)BF_4_]BF_4_, we have already observed the formation of mixtures of products^16,17^ during the abstraction of chlorine in apolar solvents with AgBF_4_. Interionic characterization of this type of complex-bearing NHC^iPr^ is studied by DFT methods (vide infra), but in order to generate active catalytic species without both chlorines, we decided to change the NHC^iPr^ ligands, using the less bulky NHC^mes^. For this purpose, [(ppy)Au(NHC^mes^)Cl]Cl was synthesized, isolated, and fully characterized (see Experimental Section for details). The characterization of the complex by mono- and bidimensional ^1^H and ^13^C NMR experiments confirms the structure of the cation previously observed for [(ppy)Au(NHC^iPr^)Cl]Cl and reported in Figure 1 together with the numbering of proton: NHC and ppy ligands are coordinated to the metal center with NHC trans to the N atom of ppy, and such ligands are perpendicular one to each other, as such a configuration around the metal center generates two different signals for the methyl groups of NHC 20 and 22 (see SI for details). The complex [(ppy)Au(NHC^mes^)OTf]OTf was generated in a NMR tube by adding 2.5 equiv of silver triflate (AgOTf) to a solution of [(ppy)Au(NHC^mes^)Cl]Cl in CD_2_Cl_2_. Characterization by multinuclear and multidimensional NMR confirms the same structure of the cation [(ppy)Au(NHC^mes^)Cl]^+^, while, in order to understand the position of the counterion around the cationic metal fragment, we recorded a ^19^F, ^1^H-HOESY NMR spectrum (Figure 3). Analysis of the ^19^F, ^1^H-HOESY NMR spectrum highlights that the anion interacts with few protons contrary to that observed for [(ppy)Au(NHC^iPr^)Cl]OTf: the strongest NOE signal is between the fluorine and the protons H1, H2, and H22. Other NOE interactions are present between OTf and the mesitylene’s H16 and H18 (Figure 3).

By normalizing all of the NOE interionic contacts (Table 2), the H1/F peak is the most intense and normalized to one (entry 5). Also, interionic contact H2/F is equal to one, even if it is important to underline that there is a superimposition with proton H7. Medium-high value (0.75) of normalized NOE intensity is found for H22 (entry 1), while lower values around 0.21–0.08 are found for H18 and H16, respectively (entries 2 and 3).

**Table 2:** Normalized (*I*/*f*) NOE Intensities for [(ppy)Au(NHC^mes^)OTf]OTf, Determined Arbitrarily by Fixing at 1 the Normalized Intensity of the NOE between the Anion and H13^a^

entry	^1^H	(*I*/*f*) NOE
1	22	0.75
2	18	0.21
3	16	0.08
4	2^b^	1.00
5	1	1.00

a The scaling factor *f* is equal to (*n*H × *n*F)/(*n*H + *n*F), where *n* is the number of magnetically equivalent nuclei (H or F).

b Partially superimposed with proton 7.

This pattern of NOE clearly indicates that one triflate is in the inner sphere in exchange in the NMR time scale with the second anion in the outer sphere. The absence of NOE signals with the imidazole ring (H13) and the phenyl of ppy (H8, H9 and H10) suggests that the second triflate is positioned near the pyridine(gold) area (Figure 1, ppy-N side and Au side) far away from gold–carbon bond of phenylpyridine (Figure 1, ppy–C-Au side). Also the counterion is located far away from the imidazole ring (Figure 1, NHC side), although this position is the most favorable in [(ppy)Au(NHC^iPr^)Cl]OTf. A possible explanation can be found in a less steric bulk of the NHC^mes^ with respect to NHC^iPr^ that makes approaching the anion near the gold atom more favorable; see the DFT section.

Another important complex formed during the pre-equilibrium step of the catalytic cycle is [(ppy)Au(NHC^iPr^)H_2_O]X_2_ in which both chlorines are replaced by X^–^ (OTf^–^ or BF_4_^–^) and water is coordinated to gold.

The complex [(ppy)Au(NHC^iPr^)H_2_O](BF_4_)_2_ was synthesized in an NMR tube by adding an excess of silver tetrafluoroborate and water to the [(ppy)Au(NHC^iPr^)Cl]Cl complex in deuterated dichloromethane.^16,17^

**Figure 4:**
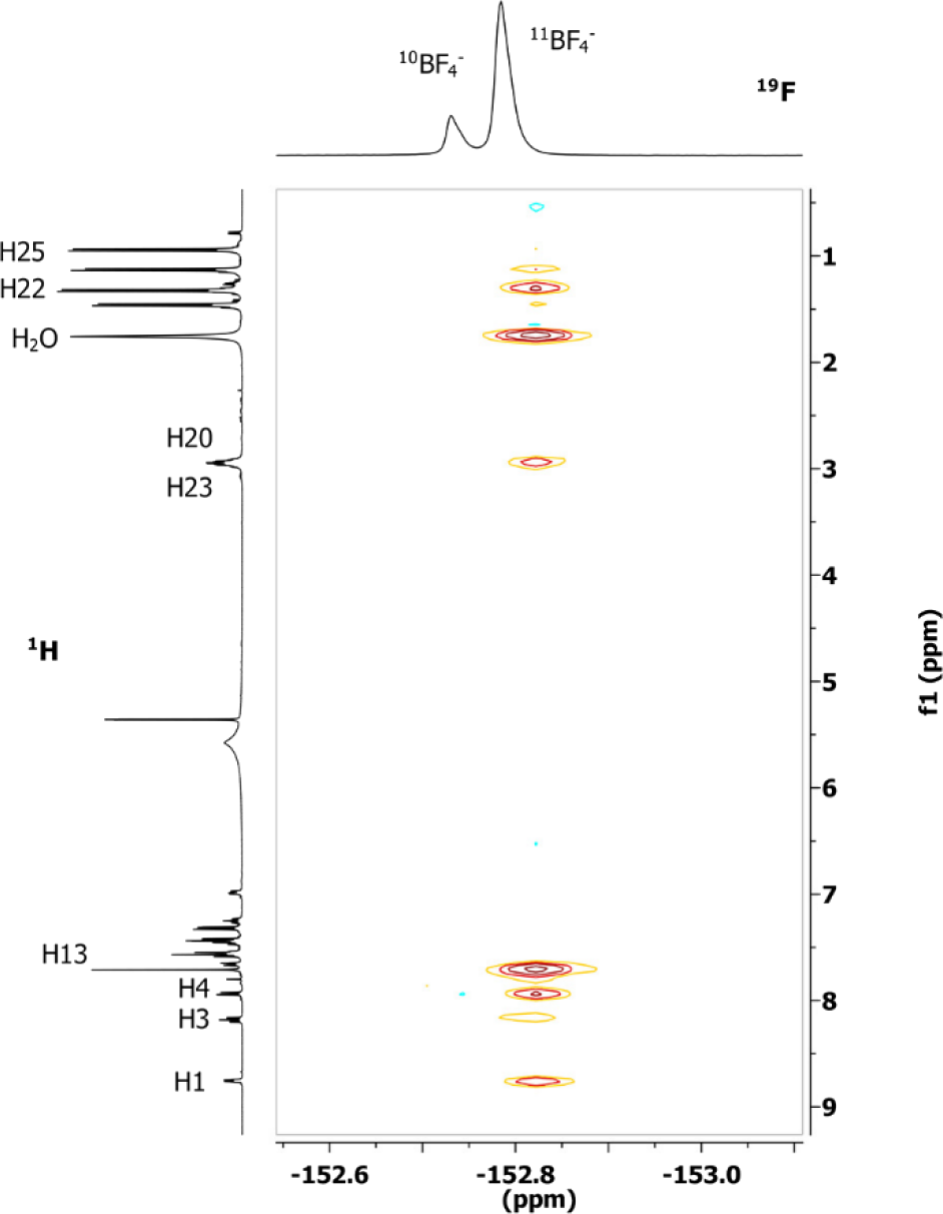
^19^F, ^1^H-HOESY NMR spectrum (376.65 MHz, 297 K, CD_2_Cl_2_) of the complex [(ppy)Au(NHC^iPr^)H_2_O](BF_4_)_2_.

Concerning the interionic structure, in the ^19^F ^1^H-HOESY NMR spectrum of [(ppy)Au(NHC^iPr^)H_2_O](BF_4_)_2_, the most intense NOE interaction between fluorine of the tetrafluoroborate anion and protons of water is observed (Figure 4 and entry 3 of Table 3). Intense NOE signal is observed with the protons of the imidazole (H13, entry 5 of Table 3), and medium/low NOE signals are also present with hydrogen of the isopropyl group (H20 and H23, entry 4 of Table 3) and methyl protons (H22, entry 2 of Table 3) that point to the imidazole ring; instead, very low NOE signal is observed with methyl protons H25 (entry 1 Table 3)

**Table 3:** Normalized (*I*/*f*) NOE Intensities for [(ppy)Au(NHC^iPr^)H_2_O](BF_4_)_2_, Determined Arbitrarily Fixing at 1 the Normalized Intensity of the NOE between the Anion and H_2_O^a^

entry	^1^H	(*I*/*f*) NOE
1	25	0.06
2	22	0.18
3	H_2_O	1.00
4	20–23	0.15
5	13	0.99
6	4	0.46
7	3	0.23
8	1	0.38

a The scaling factor *f* is equal to (*n*H × *n*F)/(*n*H + *n*F), where *n* is the number of magnetically equivalent nuclei (H or F).

Medium-strength NOE contacts were observed with the protons of the phenylpyridine: H3 and H4 (entries 6 and 7 Table 3) as observed in other complexes previously studied (Figures 2 and 3) and more interesting with H1 that point toward water molecule (Figure 4, entry 8 Table 3)

Even if the presence of two counterions can complicate the interpretation, this pattern of NOE indicates that the most favorable position of the counterions is near water (Figure 1, Y–Au side) and near the imidazole ring (Figure 1, NHC side) on the side of water. The position near the backbone of phenylpyridine is accessible by the counterion (Figure 1, ppy-N side), while the anion is located far away from the gold–carbon bond of phenylpyridine (Figure 1, ppy-C side).

The most important complex formed during the pre-equilibrium step of the catalytic cycle is [(ppy)Au(NHC^iPr^)(alkyne)]X_2_ in which both chlorines are replaced by less coordinating OTf^–^ or BF_4_^–^ and alkynes coordinate to the metal center are prompt to undergo nucleophilic attack.

Unfortunately, any attempts to synthesize, isolate, and characterize [(ppy)Au(NHC^iPr^)(3-hexyne)]X and [(ppy)Au(NHC^mes^)(3-hexyne)]X (X = OTf^–^ or BF_4_^–^) have failed in apolar and aprotic solvents.^16,17^ Interionic characterization of this type of complex is studied by DFT methods (see the next section).

### Interionic Characterization of Gold(III) Complexes by DFT Calculations

**Figure 5:**
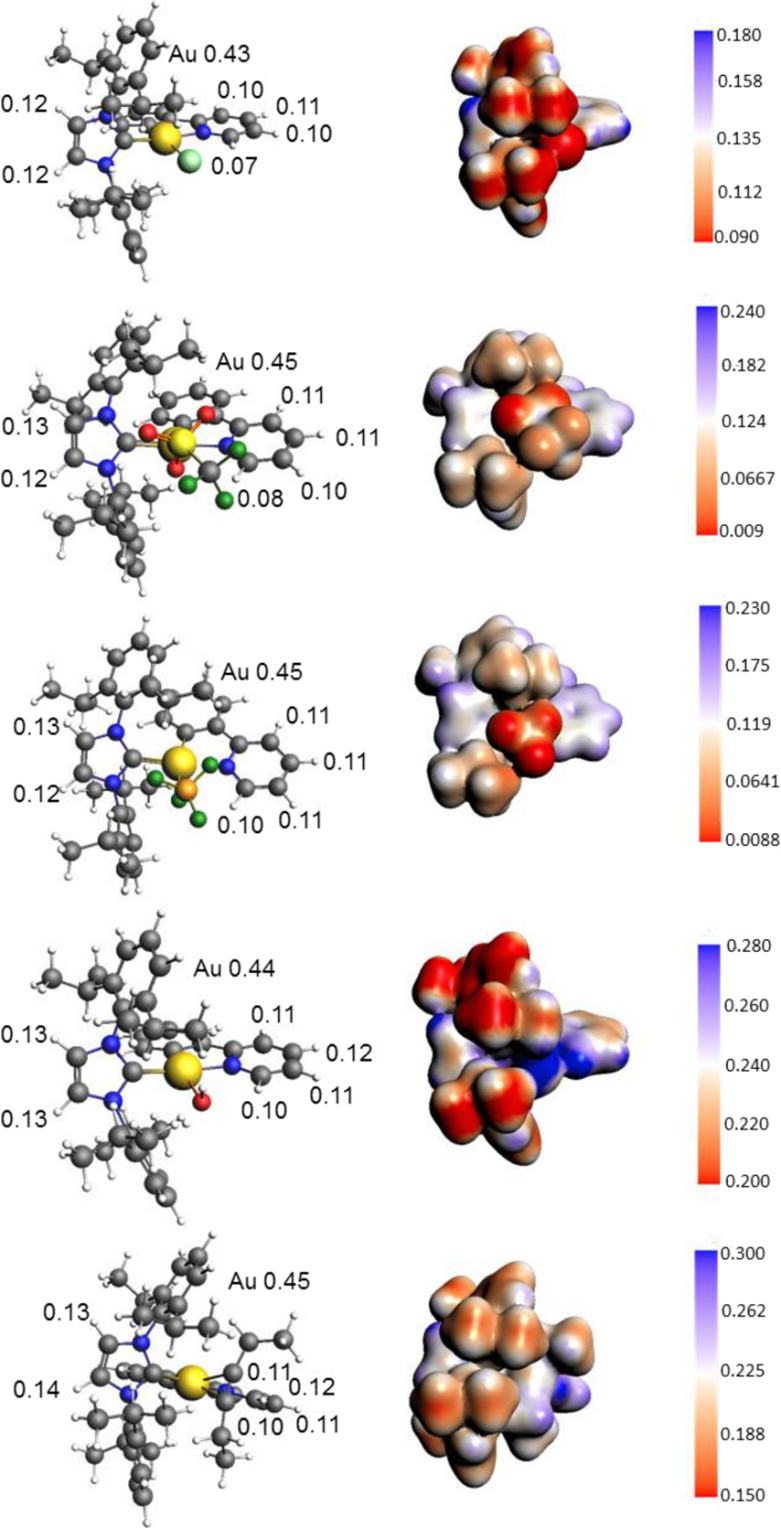
Optimized structures of [(ppy)Au(NHC^iPr^)X]^+^ (X = Cl^–^, BF_4_^–^, OTf^–^) and [(ppy)Au(NHC^iPr^)Y]^2+^ (Y = H_2_O and 3-hexyne) complexes (from top to bottom: X = Cl^–^, OTf^–^, BF_4_^–^; Y = H_2_O, 3-hexyne) and corresponding orientation views of the Coulomb potential mapped on an electronic isodensity surface (ρ = 0.007 e/Å^3^. Coulomb potential in au). Values of VDD (e) are also shown for the most attractive hydrogen atoms and for gold.

In this section, the results of DFT calculations concerning the determination of the relative anion–cation orientation(s) in [(ppy)Au(NHC^iPr^)Y]X_2_ and [(ppy)Au(NHC^iPr^)X]X (ppy = 2-phenylpyridine, X = Cl^–^, BF_4_^–^, OTf^–^; Y= H_2_O and 3-hexyne) and in the corresponding complexes with (NHC^mes^) are shown. Analysis of the Coulomb potential of the cationic complexes [(ppy)Au(NHC^iPr^)Y]^2+^, [(ppy)Au(NHC^mes^)Y]^2+^ (Y = H_2_O and 3-hexyne), and [(ppy)Au(NHC^iPr^)X]^+^, [(ppy)Au(NHC^mes^)X]^+^ (X = Cl^–^, BF_4_^–^, OTf^–^) mapped on an electronic isodensity surface is used to evaluate positively charged zones of the complex and to predict possible positions where the counterion(s) could reside in the second coordination shell. In Figure 5 these surfaces are depicted for the five considered species with (NHC^iPr^) (very similar surfaces calculated for gas phase optimized structures of the same complexes can be found in the SI, Figure S18, as expected based on the low dielectric constant of dichloromethane). Coulomb potential maps for the corresponding complexes with (NHC^mes^) are reported in the SI (Figure S19), and they show an analogous distribution of the positively charged regions of the complex as shown in Figure 5. In all the complexes, the most attractive regions on the surface (blue-colored in Figure 5) are located at the two acidic hydrogens of the imidazole ring (left side of the geometric structures, H13, see NMR intramolecular characterization, Figure 1) and at the hydrogens of ppy (right side of the geometric structures, Figure 1: H1, H2, H3). This finding suggests that the second-sphere anion would probably spend much of the time close to those hydrogens, even if in the case of H1 a negative zone due to the presence of chlorine ligand is very near and can destabilize this ion pair configuration.

One may speculate that the outer-sphere counterion could also be located close to the gold atom, formally having a positive charge. However, whereas the metal center occupies a crowded region in all the complexes bearing NHC^iPr^, where access from other species (including the counterion) is difficult for steric reasons (Figure 5), in the complexes with NHC^mes^, the accessibility of gold could be easier (Figure S19). A detailed analysis of the charge distribution on the cations carried out using the Voronoi Deformation Density (VDD) method^20^ confirms that the positive charge is significantly redistributed on the ligands. In particular, the NHC^iPr^ and ppy hydrogen atoms bear a charge of about 0.12–0.14 and 0.07–0.12 e, respectively, in all the species (Figure 5). The gold atom carries a charge ranging from 0.43 (for X = Cl^–^) to 0.45 e (for X = OTf^–^, BF_4_^–^, 3-hexyne), which is independent of the total charge of the complex (+1 or +2) (Figure 5). Analogously, the NHC^mes^ and ppy hydrogen atoms bear a charge of about 0.13–0.14 and 0.07–0.12 e, respectively, in all the species, with gold carrying a charge ranging from 0.43 (for X = Cl^–^) to 0.45 e (Figure S19).

These findings suggest that when NHC^iPr^ is replaced with NHC^mes^ the position of the anion near the gold atom can be more probable and stable, as suggested by NOE NMR experiments on [(ppy)Au (NHC^mes^)OTf]OTf (Figure 3).

The preferential position of the counterion is thus tunable through the choice of the ancillary ligands (NHC^iPr^, NHC^mes^, ppy, and Y), a result which is very analogous to that obtained for the Au(I) cationic complexes of general formula [L-Au(I)^+^···X^–^] (L = phosphines or NHCs, X^–^ = weakly coordinating anion).

**Figure 6:**
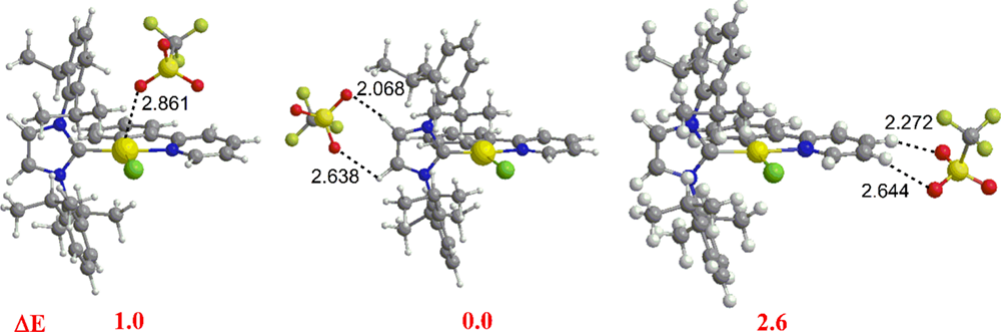
Optimized structures of [(ppy)Au(NHC^iPr^)Cl]OTf ion pairs: anion close to gold (OTf–Au, orange region of Figure 1, Au side) (left), to H13 acidic hydrogen atoms of NHC^iPr^ (OTf–H13, red region of Figure 1, NHC side) (middle) and to ppy acidic hydrogens H2 and H3 (OTf–H2/H3, blue region of Figure 1, ppy-N side) (right). Relevant distances (Å) and relative energies (Δ*E* in kcal/mol) of ion pairs are also shown.

**Figure 7:**
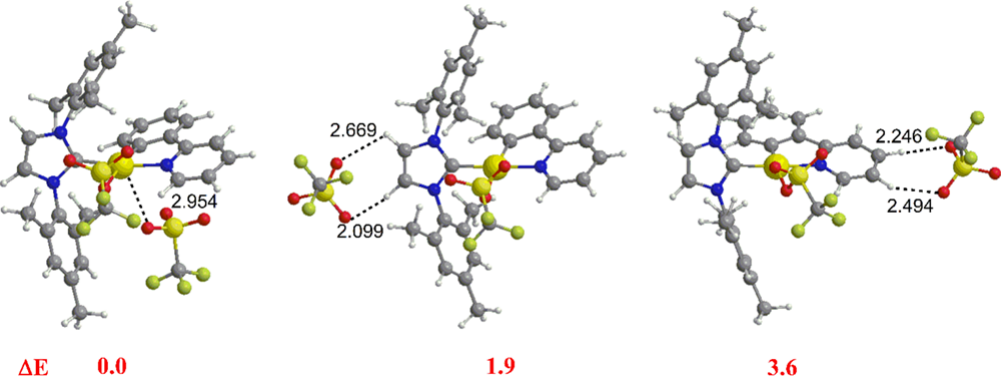
Optimized structures of [(ppy)Au(NHC^mes^)OTf]OTf ion pairs: anion close to gold (OTf–Au, orange region of Figure 1, Au side) (left), to H13 acidic hydrogen atoms of NHC^mes^ (OTf–H13, red region of Figure 1, NHC side) (middle) and to ppy acidic hydrogens H2 and H3 (OTf–H2/H3, blue region of Figure 1, ppy-N side) (right). Relevant distances (Å) and relative energies (Δ*E* in kcal/mol) of the ion pairs are also shown.

To further analyze the ion pairing, geometry optimizations have been carried out for three different configurations of [(ppy)Au(NHC^iPr^)Cl]OTf and [(ppy)Au(NHC^mes^)OTf]OTf, selected as a prototype of all the complexes, where the counterion is placed close to the H13 of NHC^iPr^ (NHC^mes^) to the ppy ligands and to the gold center. Configuration structures and their relative energies are shown in Figures 6 and 7.

The most stable configuration for the [(ppy)Au(NHC^iPr^)Cl]OTf ion pairs is that where OTf^–^ is located at the NHC^iPr^ (OTf–H13, red region of Figure 1, NHC side) (Figure 6, middle), consistent with the experimental results (Figure 2). However, structures where the counterion is positioned close to the metal center (OTf–Au, orange region of Figure 1, Au side; Figure 6, left) and ppy (OTf–H2/H3, blue region of Figure 1, ppy-N side; Figure 6, right) are less stable by only 1.0 and 2.6 kcal/mol, respectively, indicating a flat PES for the three configurations.

For the [(ppy)Au(NHC^mes^)OTf]OTf ion pairs, the most stable configuration is that where OTf^–^ is close to the metal center (OTf–Au) (Figure 7, left), in agreement with the experiment (Figure 3), whereas configurations where the counterion is located at the (NHC^mes^) side (OTf–H13) (Figure 7, middle) and at the ppy side (OTf–H2/H3) (Figure 7, right) are less stable by 1.9 and 3.6 kcal/mol, respectively, analogously indicating a flat PES for the three configurations. These results show that a one-to-one mapping between the Coulomb potential and the anion electrostatic interaction cannot be found and suggest that the less steric bulk of the NHC^mes^ with respect to NHC^IPr^ makes more favorable approaching the anion near the gold atom. Indeed, a similar analysis of the three ion pairs performed for the [(ppy)Au(NHC^mes^)Cl]OTf complex confirms that the most stable configuration is that where OTf^–^ is close to the gold center and that a flat PES for the three configurations is involved (Figure S20 in the SI). One may expect that noncovalent interactions between OTf^–^ (at the three different positions) and the surrounding environment could influence the relative stability of the three ion pair conformations. To assess the effects of the solvent and the method used for the inclusion of dispersion correction on the relative stability of the three conformations, a few test calculations have been carried out. Results are reported in the SI (Figure S21 and Tables S2 and S3). As expected, the solvent affects the geometries by generally increasing the OTf–H13 and OTf–H2/H3 distances. However, the PES for the three different conformations is not significantly influenced by the solvent as well as by the method used for dispersion correction inclusion, and it remains flat (see Figure S21 and Tables S2 and S3, i.e., the three conformations lie very close in energy).

Finally, to get an estimate of how easy the replacement of coordinated Cl^–^ by OTf^–^ and BF_4_^–^ is, the binding energy of the different anions to gold have been computed for [(ppy)Au(NHC^iPr^)]^2+^ as an example. Results show that the gold complex has the highest bonding energy with Cl^–^ (Δ*G* = −44.0 kcal/mol), whereas with OTf^–^ and BF_4_^–^ the bonding energies are significantly lower (−17.5 and −12.5 kcal/mol, respectively), thus suggesting that Cl^–^ replacement by OTf^–^ and BF_4_^–^ is thermodynamically unfavorable, consistent with the experimental data (Table S14 in the SI).

## Conclusions

We have shown that the combination of experimental results coming from interionic NOE NMR studies and theoretical DFT calculations of the Coulomb potential provides a reliable methodology for disclosing the relative orientation of the anion and cation within an ion pair.^21^ We have applied such a methodology to the investigation of gold complexes [(ppy)Au(NHC)Y]X_2_ and [(ppy)Au(NHC)X]X that change in the nature of the carbenes (NHC^iPr^ versus NHC^mes^), Y (H_2_O and 3-hexyne), and counterion (Cl^–^, BF_4_^–^ and OTf^–^). Two main orientations have been observed: ppy-N side and NHC-side, even if the exact position of the anion is finely modulated by NHC and Y. When Y = Cl and NHC = NHC^iPr^, the counterion also approaches the Au side and ppy-C side as confirmed by DFT calculations that indicate a flat PES with different conformations lying closer in energy. In fact, the positive charge, formally localized on the gold atom, is strongly delocalized over the whole of the complexes as indicated by the VDD analysis and maps of electrostatic potentials obtained by DFT calculations.

In contrast, when Y = H_2_O, it is found that the introduction of the rather acidic H protons of water causes a decrease of the abundance of the ppy-N side and NHC-side orientations in favor of Y side: coordination of H_2_O increases the positive charge on H, giving the anion a very good anchoring point. Also, the nature of NHC changes the interionic structure: when changing NHC^iPr^ to less encumbered NHC^mes^ the position near gold (Au side) becomes more accessible and the position near imidazole ring (NHC side) becomes less favored.^22^

Finally, DFT calculations indicate that in [(ppy)Au(NHC)(3-hexyne)]X_2_ activated complexes that undergo nucleophilic attack, the counterion can approach NHC side, ppy-N side, and Au side (Figure 1). From these positions, the anion can still acts as a template, holding water in the right position for the outer-sphere attack.^16,17^

In conclusion, the preferential position of the counterion is tunable through the choice of the ancillary ligands (NHC^iPr^, NHC^mes^, ppy, and Y) and it is remarkable that such effects deriving from differences in energy smaller than 1 kcal/mol could be clearly detected by NMR and completely rationalized with the help of DFT calculations. However, rationalization of counterion effects in cationic gold(III) catalysis is still in its infancy. The absence of a comprehensive mechanistic understanding has hindered the rational selection of counterions in gold(III)-catalyzed reactions. Further mechanistic studies and novel control experiments are needed for a deeper understanding of the counterion’s role in gold(III) catalysis.^23^

## Experimental Section ad Computational Details

### Synthesis and Intramolecular Characterization

Silver triflate (AgOTf), silver tetrafluoroborate (AgBF4), and KHCO_3_ were purchased from Sigma-Aldrich. All the solvents were used as received without any further purification unless otherwise stated. 1,3-bis(diisopropylphenyl)imidazolium chloride (NHC^iPr^·HCl), 1,3-bis(2,4,6-trimethylphenyl)-imidazolium chloride (NHC^mes^·HCl),^24^ and dichlorophenylpyridinegold(III) [AuCl_2_(ppy)]^25^ were synthesized according to the literature. All compounds were characterized in solution by ^1^H, ^13^C, ^19^F, and ^14^N NMR spectroscopies. NMR spectra were recorded on an Avance 400 III HD spectrometer. Chemical shifts (ppm) are relative to TMS for both ^1^H and ^13^C nuclei, whereas ^31^P, ^19^F, and ^15^N chemical shifts are referenced to 85% H_3_PO_4_, CCl_3_F, and CH_3_NO_2_, respectively. The complexes were fully characterized with monodimensional (^1^H and ^13^C) and bidimensional (^1^H–^1^H COSY, ^1^H–^1^H NOESY, ^1^H–^13^C HSQC, ^1^H–^13^C HMBC, ^1^H–^15^N HMBC) NMR experiments.

### Synthesis and Intramolecular Characterization of [(ppy)Au(NHC^iPr^)Cl]BF_4_

The complex [(ppy)Au(NHC^iPr^)Cl]BF_4_ was synthesized with the same procedure adopted to prepare [(ppy)Au(NHC^iPr^)Cl]OTf:^16,17^ 20 μmol of silver triflate (1.1 equiv) was added to 15 mg (18.5 μmol) of [(ppy)Au(IPr)Cl]Cl in 5 mL of dichloromethane. The solution was filtered on Celite and the volume of the solution was then reduced. The desired compound was precipitated by the addition of pentane. Yield was 92%.

^1^H NMR (400 MHz. CD_2_Cl_2_. 298 K): δ(ppm) = 9.35 (dd. 1H. ^3^*J*_HH_ = 6.1. ^4^*J*_HH_ = 1.6 Hz. H1). 8.18 (td. 1H. ^3^*J*_HH_ = 7.8. ^4^*J*_HH_ = 1.6 Hz. H3). 7.98 (dt. 1H. ^3^*J*_HH_ = 8.1. ^4^*J*_HH_ = 1.1 Hz. H4). 7.80 (s. 2H. H13). 7.75 (dd. 1H. ^3^J_HH_ = 7.9. ^4^*J*_HH_ = 1.6 Hz. H7). 7.60–7.49 (m. 4H. H2–8–17). 7.41 (dd. 2H. ^3^*J*_HH_ = 7.8. ^4^*J*_HH_ = 1.5 Hz. H18). 7.36 (td. 1H. ^3^*J*_HH_ = 7.7. ^4^*J*_HH_ = 1.6 Hz. H9). 7.30 (dd. 2H. ^3^*J*_HH_ = 7.8. ^4^*J*_HH_ = 1.5 Hz. H16). 7.02 (dd. 1H. ^3^*J*_HH_ = 7.8. ^4^*J*_HH_ = 1.1 Hz. H10). 3.07 (hept. 2H. ^3^*J*_HH_ = 6.7 Hz. H20). 2.92 (hept. 2H. ^3^*J*_HH_ = 6.7 Hz. H23). 1.48 (d. 6H. ^3^*J*_HH_ = 6.6 Hz. H24). 1.27 (d. 6H. ^3^*J*_HH_ = 6.7 Hz. H22). 1.15 (d. 6H. ^3^*J*_HH_ = 6.8 Hz. H25). 0.79 (d. 6H. ^3^*J*_HH_ = 6.7 Hz. H21).

^19^F NMR (400 MHz. CD_2_Cl_2_. 298 K): δ = −152.98 – −153.09 (m).

### Synthesis and Intramolecular Characterization of [(ppy)Au(NHC^mes^)Cl]Cl

The complex was synthesized according to the literature procedure.^16,17^ In a Schlenk flask, 1 equiv (0.25 mmol) of NHC^mes^·HCl, 1.1 equiv (116.1 mg, 0.275 mmol) of [AuCl_2_(ppy)], and 4 equiv (100.1 mg, 1 mmol) of KHCO_3_ were dissolved in 10 mL of acetonitrile and stirred at room temperature overnight. The solvent was then removed under vacuum, the residue was dissolved in dichloromethane (10 mL), and the mixture was filtered through a paddle of Celite. The volume of the solution was reduced to about 2 mL and the complex was precipitated by adding *n*-pentane. The white microcrystalline product was collected by filtration, washed with *n*-pentane (2 × 2 mL), and dried under vacuum. Yield was 94%.

^1^H NMR (400 MHz. CDCl_3_. 298 K): δ(ppm) 9.33 (dd. 1H. ^3^*J*_HH_ = 6.0 Hz. ^4^*J*_HH_ = 1.6 Hz. H1). 8.40 (dd. 1H. ^3^*J*_HH_ = 8.1 Hz. ^4^*J*_HH_ = 1.6 Hz. H4). 8.34 (td. 1H. ^3^*J*_HH_ = 7.7 Hz. ^4^*J*_HH_ = 1.5 Hz. H3). 7.96 (m. 3H. H7–13). 7.56–7.44 (m. 2H. H2–8). 7.25 (td. 1H. ^3^*J*_HH_ = 7.7 Hz. ^4^*J*_HH_ = 1.5 Hz. H9). 7.01 (dd. 2H ^3^*J*_HH_ = 2.0 Hz. ^4^*J*_HH_ = 1.1 Hz. H18). 6.91 (dd. 1H. ^3^*J*_HH_ = 7.9 Hz. ^4^*J*_HH_ = 1.1 Hz. H10). 6.86 (s. 2H. H16). 2.33 (s. 6H. H22). 2.28 (s. 6H. H21). 2.24 (s. 6H. H20).

^13^C {^1^H} NMR (101 MHz. CDCl_3_. 298 K): δ(ppm) 163.84 (1C. C5). 148.34 (1C. C12). 147.72 (1C. C1). 147.52 (1C. C6). 144.30 (1C. C3). 143.03 (1C. C11). 140.86 (2C. C17). 136.19 (2C. C19). 134.26 (1C. C10). 133.35 (1C. C15). 132.87 (2C. C14). 131.53 (1C. C9). 130.40 (2C. C18). 130.27 (1C. C8). 130.05 (2C. C16). 127.52 (2C. C13). 127.31 (1C. C7). 124.44 (1C. C2). 122.38 (1C. C4). 21.06 (2C. C21). 19.74 (2C. C20). 19.49 (2C. C22).

^15^N NMR (41 MHz. CDCl_3_. 298 K): δ(ppm) −147.37 (1N. N1). −187.87 (2N. N2).

### Synthesis and Intramolecular Characterization of [(ppy)Au(NHC^mes^)OTf]OTf

The complex [(ppy)Au(NHC^mes^)OTf]OTf was synthesized in a NMR tube adding 2.5 equiv of AgOTf to 20 mg of [(ppy) Au NHC^mes^ Cl]Cl in CDCl_3_. Attempts to isolate the complex have shown its instability in air and temperature.

^1^H NMR (400 MHz. CDCl_3_. 298 K): δ(ppm) 8.72 (d. 1H. ^3^*J*_HH_ = 5.9 Hz. H1). 8.09 (td. 1H. ^3^*J*_HH_ = 7.9. ^4^*J*_HH_ = 1.4 Hz. H3). 7.83 (d. 1H. ^3^*J*_HH_ = 8.0 Hz. H4). 7.62 (s. 2H. H13). 7.61–7.51 (m. 2H. H2–7). 7.43 (td. 1H. ^3^*J*_HH_ = 7.6. ^4^*J*_HH_ = 1.0 Hz. H8). 7.21–7.08 (m. 3H. H9–18). 7.18 (s. 2H. H18). 6.94 (s. 2H. H16). 6.75 (dd. 1H. ^3^*J*_HH_ = 8.1. ^4^*J*_HH_ = 1.1 Hz. H10). 2.32 (s. 6H. H21). 2.29 (s. 6H. H22). 2.23 (s. 6H. H20).

^13^C NMR (101 MHz. CDCl_3_. 298 K) δ(ppm) 160.23 (1C. C5). 149.74 (1C. C1). 147.21 (1C. C12). 143.68 (1C. C3). 143.11 (1C. C11). 141.45 (2C. C17). 136.57 (1C. C6). 135.39 (2C. C19). 134.33 (1C. C10). 132.57 (2C. C15). 131.77 (2C. C14). 131.47 (2C. C18). 130.88 (2C. C8–9). 130.23 (2C. C16). 126.66 (3C. C7–13). 126.14 (1C. C2). 120.80 (1C. C4). 21.04 (2C. C21). 19.29 (2C. C20). 18.31 (2C. C22).

^15^N NMR (41 MHz. CDCl_3_. 298 K) δ(ppm) −142.68 (1N. N1). −186.71 (2N. N2).

^19^F NMR (376 MHz. CDCl_3_. 298 K): δ(ppm) −78.10 ppm.

### NOE Measurements

Two-dimensional ^19^F, ^1^H-HOESY NMR experiments were acquired using the standard four-pulse sequence.^26^ The number of transients and data points was chosen according to the sample concentration and the desired final digital resolution. Semiquantitative spectra were acquired by using a 1-s relaxation delay and 800 ms mixing times.

## Computational Details

DFT calculations have been performed using the Amsterdam Density Functional (ADF) (2014 version)^27−29^ and the related Quantum-regions Interconnected by Local Descriptions (QUILD)^30^ program packages. For geometry optimization, the GGA BP86 functional^31,32^ and a Slater-type TZ2P triple-ζ basis set with two polarization functions for all atoms, in the small frozen core approximation, were used. Relativistic effects were included by the scalar zero-order regular approximation ZORA Hamiltonian^33−35^ The Grimme 3 BJDAMP dispersion correction^36^ and solvation, by the Conductor like Screening Model COSMO,^37−39^ using dichloromethane as a solvent, were also included in optimization calculations (BP86-D3BJ1.0 COSMO). Explicit evaluation of the dispersion forces contribution to the relative energy of the ion pairs has been carried out by comparing three different models (Grimme 3 BJDAMP with S6 = 1.0, Grimme 3 BJDAMP with S6 = 0.64^40^ and Grimme 3, see Table S3 in the SI). Assessment of the solvation effect on the geometry has also been done (see Figures S18 and S21 and Table S2 in the SI). Relative energies of the different configurations have been computed by single-point Grimme 3 BJDAMP with S6 = 0.64 calculations performed on the optimized BP86-D3BJ1.0 COSMO structures (Figures 6 and 7).

## Supporting Information Available

Synthesis, Intramolecular, and Interionic Characterization by NMR Experiments of Gold(III) Complexes (ppy)Au(NHC^iPr^)Cl]BF_4_ (Figure S1), [(ppy)Au(NHC^mes^)Cl]Cl (Figures S2–S9), [(ppy)Au(NHC^mes^)OTf]OTf (Figures S10–S17 and Table S1), DFT calculations (Figures S17–S20 and Table S1): Evaluation of the dispersion correction and solvent effects on the relative stability of the ion pair conformations (Figure S21 and Tables S2-S3), Evaluation of the coordination ability of [(ppy)Au(NHC^iPr^)]^2+^ towards Cl^–^, OTf^–^ and BF_4_^–^ (Table S4) (PDF)
Cartesian coordinates for the calculated structure of complex (XYZ)

## Supplementary Material

om3c00293_si_001.pdf

om3c00293_si_002.xyz
