# Supplementary material for: Experimental and Theoretical Investigation of Ion Pairing in Gold(III) Catalysts
Source: Organometallics. 2023 Sep 30;42(20):2973–82. doi: 10.1021/acs.organomet.3c00293 (PMC10599130; doi:10.1021/acs.organomet.3c00293)
Supplement: Supplementary file 1 — om3c00293_si_001.pdf [file om3c00293_si_001.pdf]

Experimental and theoretical investigation of Ion pairing in gold (III) catalysts

Jacopo Segato,[a] Eleonora Aneggi,[a], Walter Baratta, [a], Filippo Campagnolo,[a] Leonardo Belpassi,[b] Paola Belanzoni,[b][c], Daniele Zuccaccia,[a]**
[a] Dipartimento di Scienze Agroalimentari, Ambientali e Animali, Sezione di Chimica, Università di Udine, Via Cotonificio 108, I-33100 Udine, Italy. E-mail: daniele.zuccaccia@uniud.it
[b] Istituto di Scienze e Tecnologie Chimiche (SCITEC), Consiglio Nazionale delle Ricerche c/o Dipartimento di Chimica, Biologia e Biotecnologiee, Università degli Studi di Perugia, Via Elce di Sotto 8, 06123 Perugia, Italy
[c] Department of Chemistry, Biology and Biotechnology, University of Perugia, via Elce di Sotto 8, 06123 Perugia, Italy. E-mail: paola.belanzoni@unipg.it

SUPPORTING INFORMATION

SUMMARY:

1. NMR INTRAMOLECULAR AND INTERIONIC CHARACTERIZATION…..S2
2. DFT CALCULATIONS…………………………………………………..….S22

1. Intramolecular and Interionic characterization

Synthesis and intramolecular characterization of [(ppy)Au(NHC^{iPr})Cl]BF_4

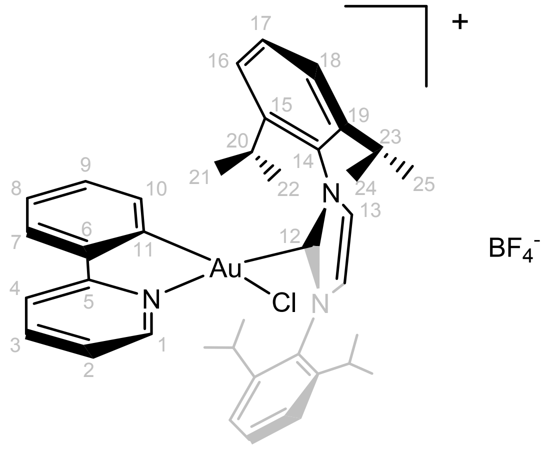

The ^{1}H NMR signals of the [(ppy)Au(NHC^{iPr})Cl]BF_4 in deuterated dichloromethane is quite similar to the [(ppy)Au(NHC^{iPr})Cl]Cl in the same solvent.[1] The iso-propyls do not present any significant shift compared to the dichloride, while in the aromatic area there are few differences. The most significant variations are for the proton of the phenylpyridine H4 and H7 with 0.20 ppm (from 8.18 to 7.98) and 0.14 ppm (from 7.89 to 7.75 ppm) respectively. While for the imidazole the two protons H13 shifted of 0.17 ppm from 7.97 vs 7.80 ppm.

Analyzing the ^{1}H-^{19}F HOESY NMR spectrum (Figures S1a and S1b) it is evident that the anion interacts with the proton H4 of the phenylpyridine ligand and with the protons H13, H22 and H25 of the NHC ligand. Interesting BF_4 does not interact with H1 and H24, the protons are located close to the chlorine. The most intense signals are with the protons of the imidazole (H13) and with the methyl protons (H22 and H25) that point to the imidazole ring. Medium-strength contacts were observed with the protons of the phenylpyridine (H4) which face to the opposite side of the cyclometalated.

There is a clear indication from the NOE that the counterion favourite positions are near the imidazole ring (NHC side) and ppy-N side, ad observed for [(ppy)Au(NHC^{iPr})Cl]OTf

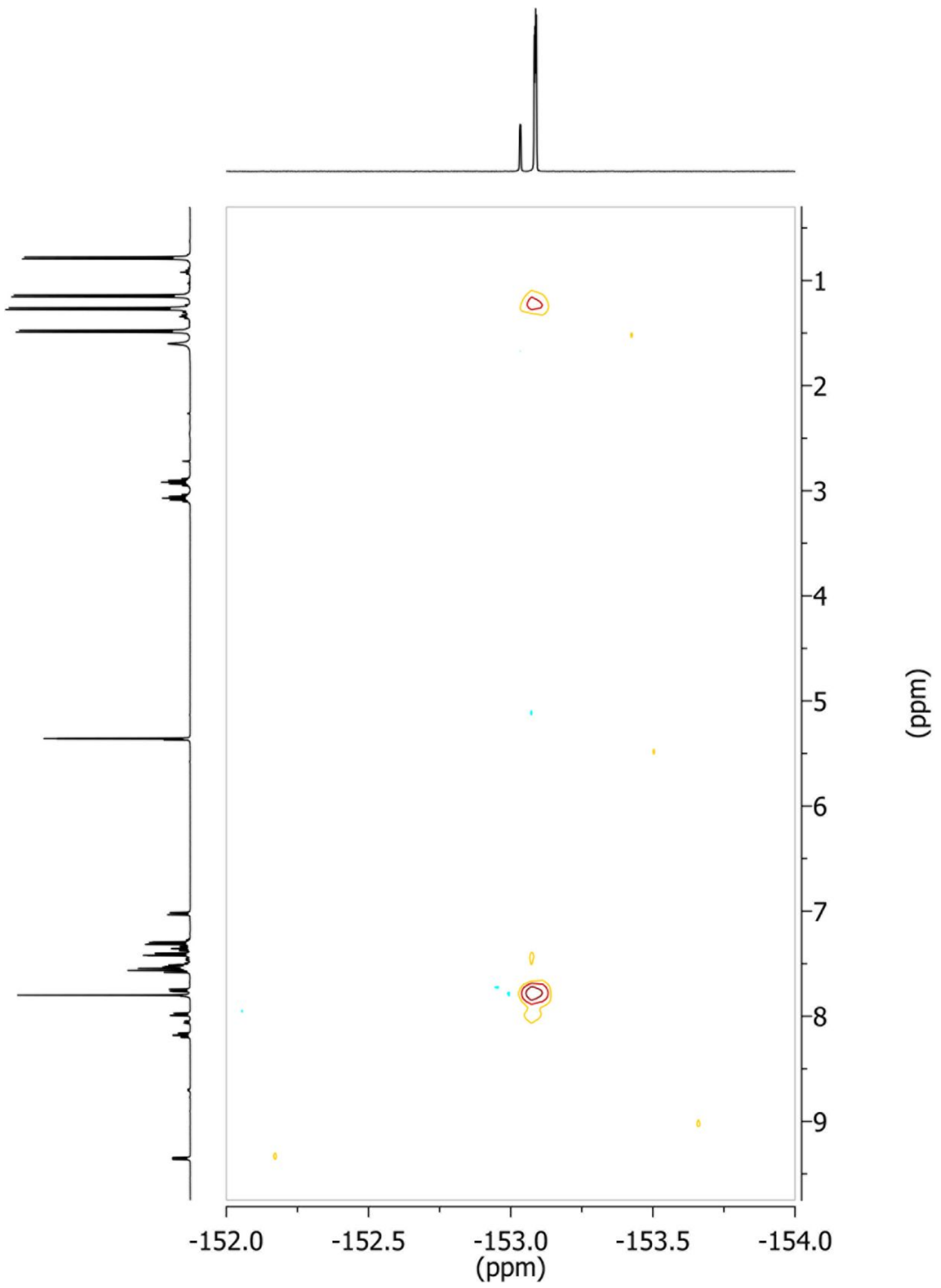

Figure S1a. ^{19}F,^{1}H-HOESY NMR spectrum (376.65 MHz, 297K, CD_2Cl_2) of complex $[(ppy)Au(NHC^{iPr})Cl]BF_4$.

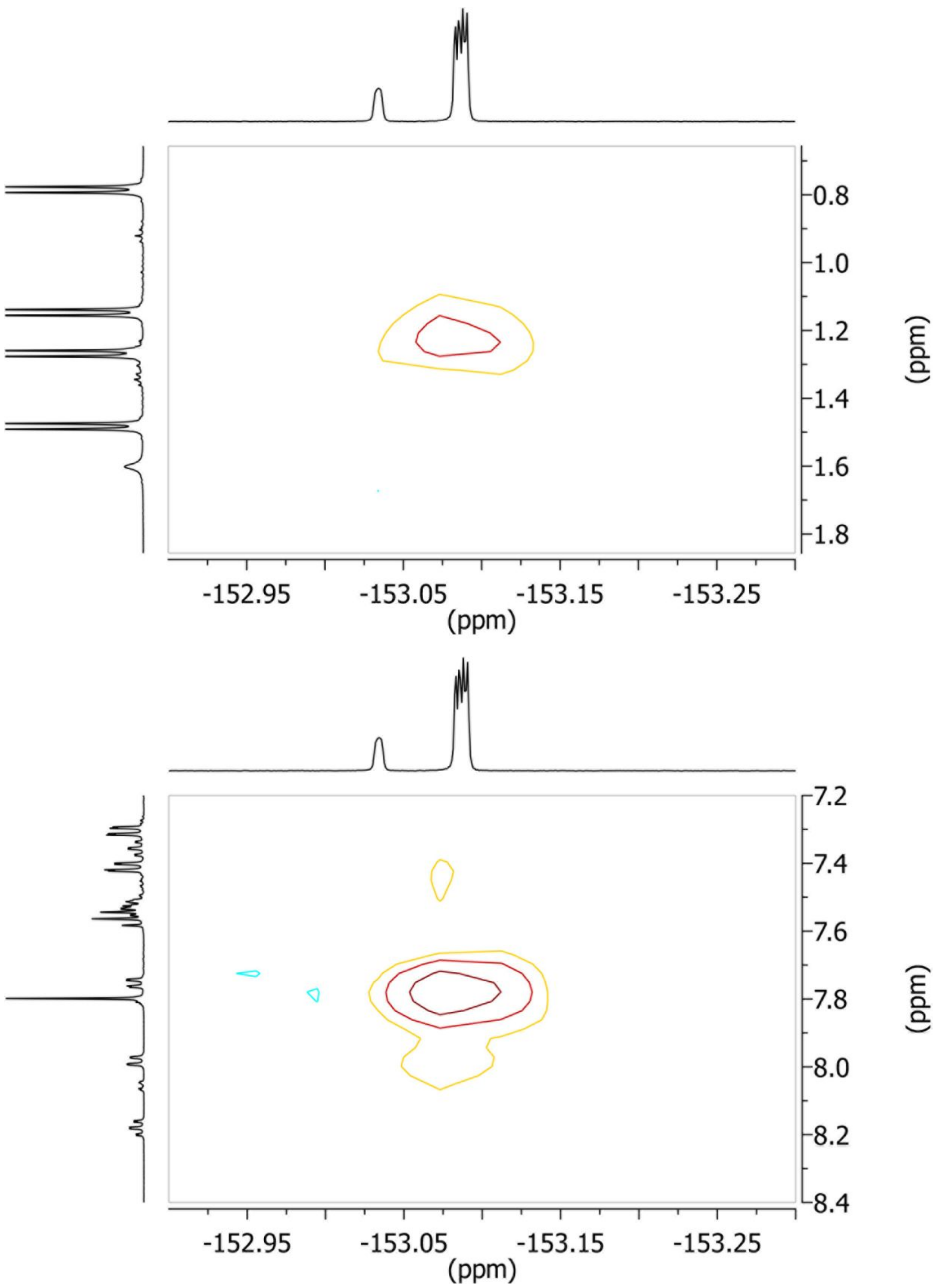

Figure S1b. A sections of $^{19}F,^{1}H$-HOESY NMR spectrum (376.65 MHz, 297K, CD_2Cl_2) of complex $[(ppy)Au(NHC^{iPr})Cl]BF_4$.

Synthesis and intramolecular characterization of [(ppy)Au(NHC^{mes})Cl]Cl

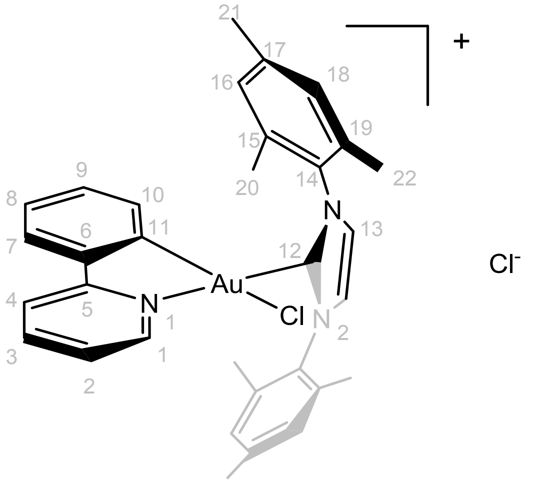

The proton close to the nitrogen of the phenylpyridine is the most de-shield and it presents a doublet at 9.33 ppm (H1), while the hydrogen most shield is the one close to the carbon bonded to gold at 6.91 ppm (H10), Figure S2. The higher chemical-shift was chosen as starting point to enumerate the molecule and assign the protons shifts. Integration confirm that the number of protons correspond to the expected structure. For what concern the NHC^{mes} the two imidazole protons are the most de-shielded, and due to a symmetry plane through the five member-ring, the two signals present an intense singlet at 7.96 ppm. In the aliphatic region are presents three singlets at 2.33. 2.28 and 2.24 ppm corresponding to the methyl groups of the mesitylene rings.

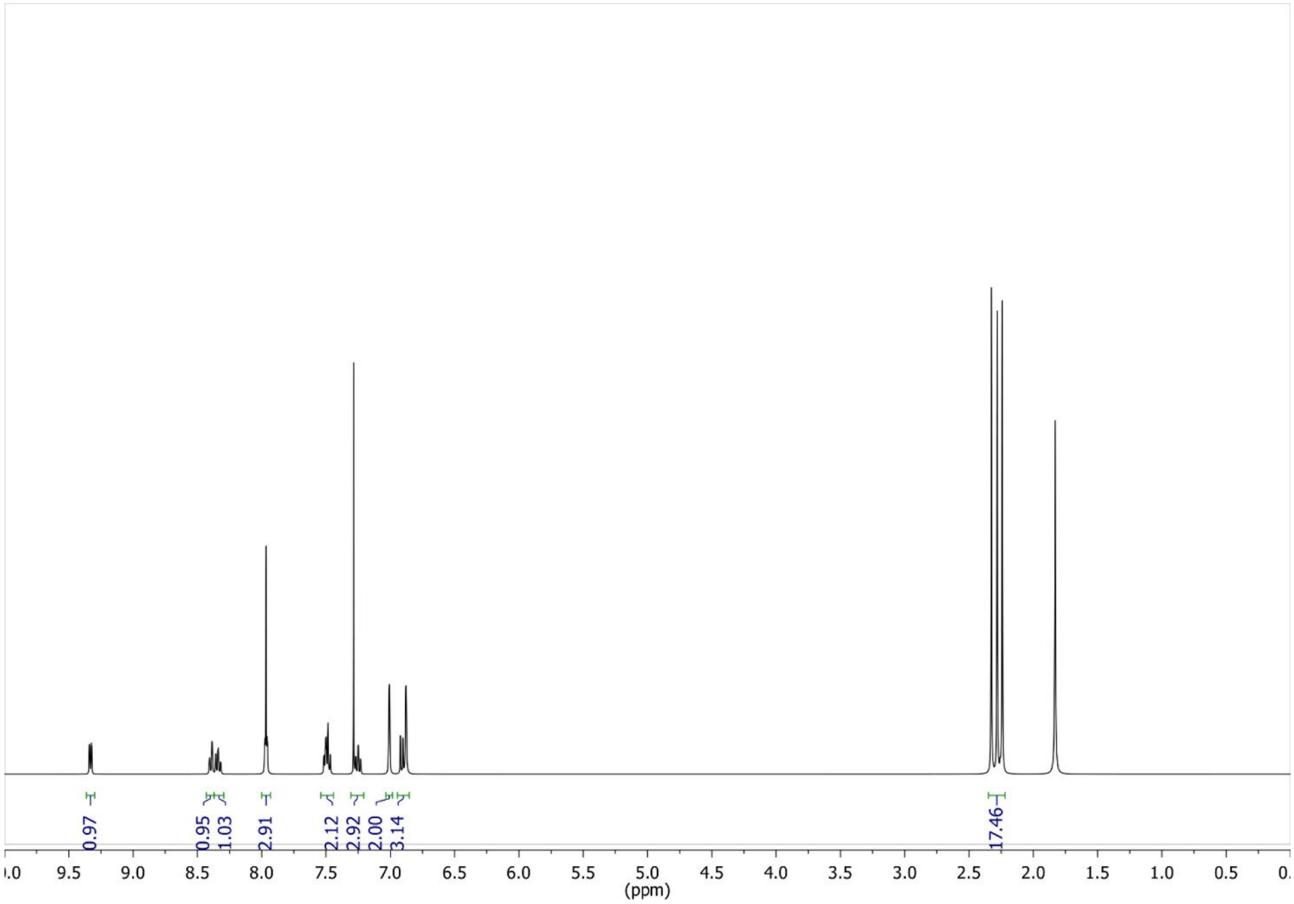

Figure S2. ^{1}H NMR spectrum (376.65 MHz, 297K, $CDCl_3$) of complex [(ppy)Au(NHC^{mes})Cl]Cl

The sequence of the protons in the phenylpyridine rings was determined with the help of the ^{1}H-^{1}H COSY experiment, Figure S3. H4 and H3 rise a doublet and a triplet at 8.40 and 8.34 ppm. The H7 doublet is under the imidazole H13 signals at 7.96. H2 and H8 form a multiplet between 7.56 and 7.44 ppm. H9 is the triplet close to the no-deuterated signal of the solvent at 7.25 ppm. In the aliphatic area H21 is the only signal which can correlate with both the aromatic protons H16 and H18 and it is the singlet at 2.28 ppm.

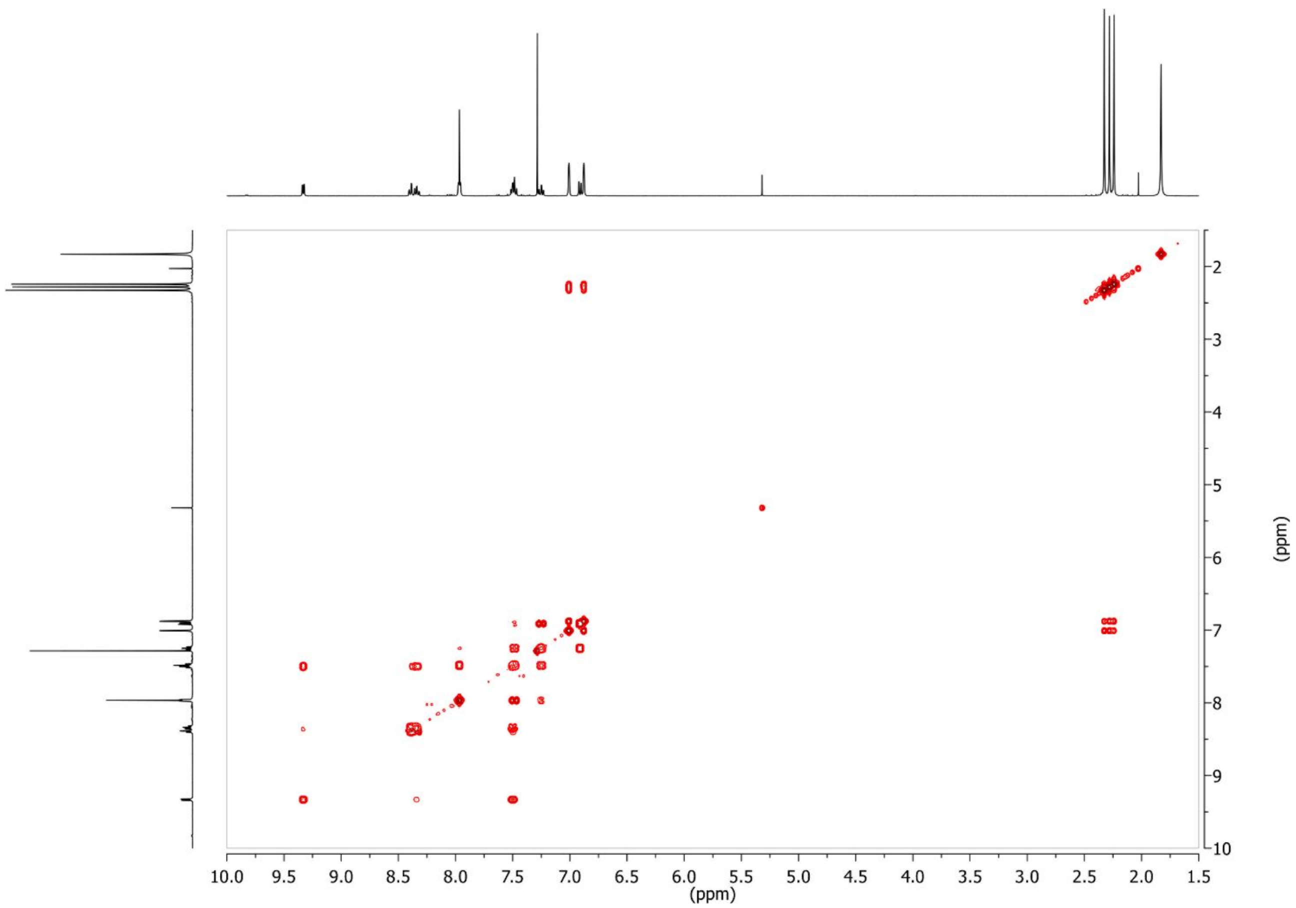

Figure S3. ^{1}H-^{1}H COSY NMR spectrum (376.65 MHz, 297K, $CDCl_3$) of complex [(ppy)Au(NHC^{mes})Cl]Cl

The remaining signals were attributed with the help of ^{1}H-^{1}H NOESY experiment. The signal at 2.24 ppm gives an Overhouse Effect with the protons at 9.91 labelled as H10 (Figures S4 and S5). This singlet therefore is the methyl group number as H20. The third singlet at 2.33 ppm must be the H22. The H16 and H18 are the singlet which integrate 2 protons corresponding at 6.86 and 7.01 ppm.

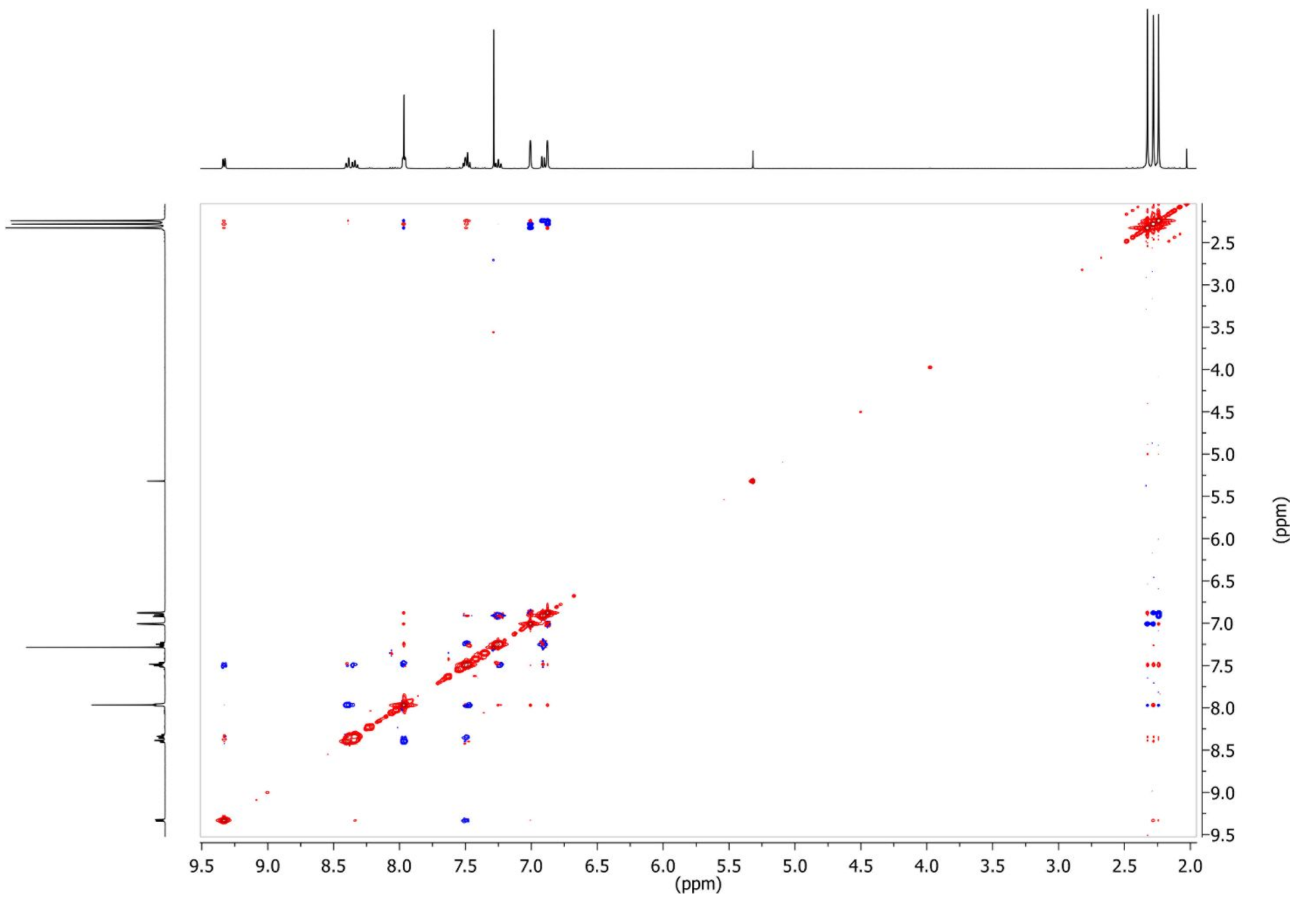

Figure S4. ^{1}H-^{1}H NOESY NMR spectrum (376.65 MHz, 297K, CD_2Cl_2) of complex [(ppy)Au(NHC^{mes})Cl]Cl

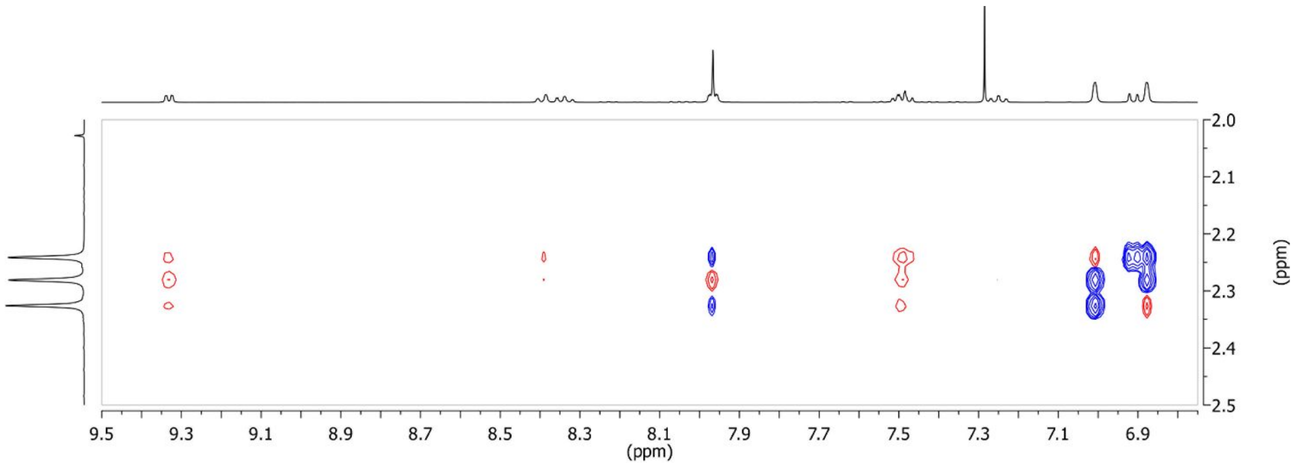

Figure S5. A section of ^{1}H-^{1}H NOESY NMR spectrum (376.65 MHz, 297K, $CDCl_3$) of complex [(ppy)Au(NHC^{mes})Cl]Cl

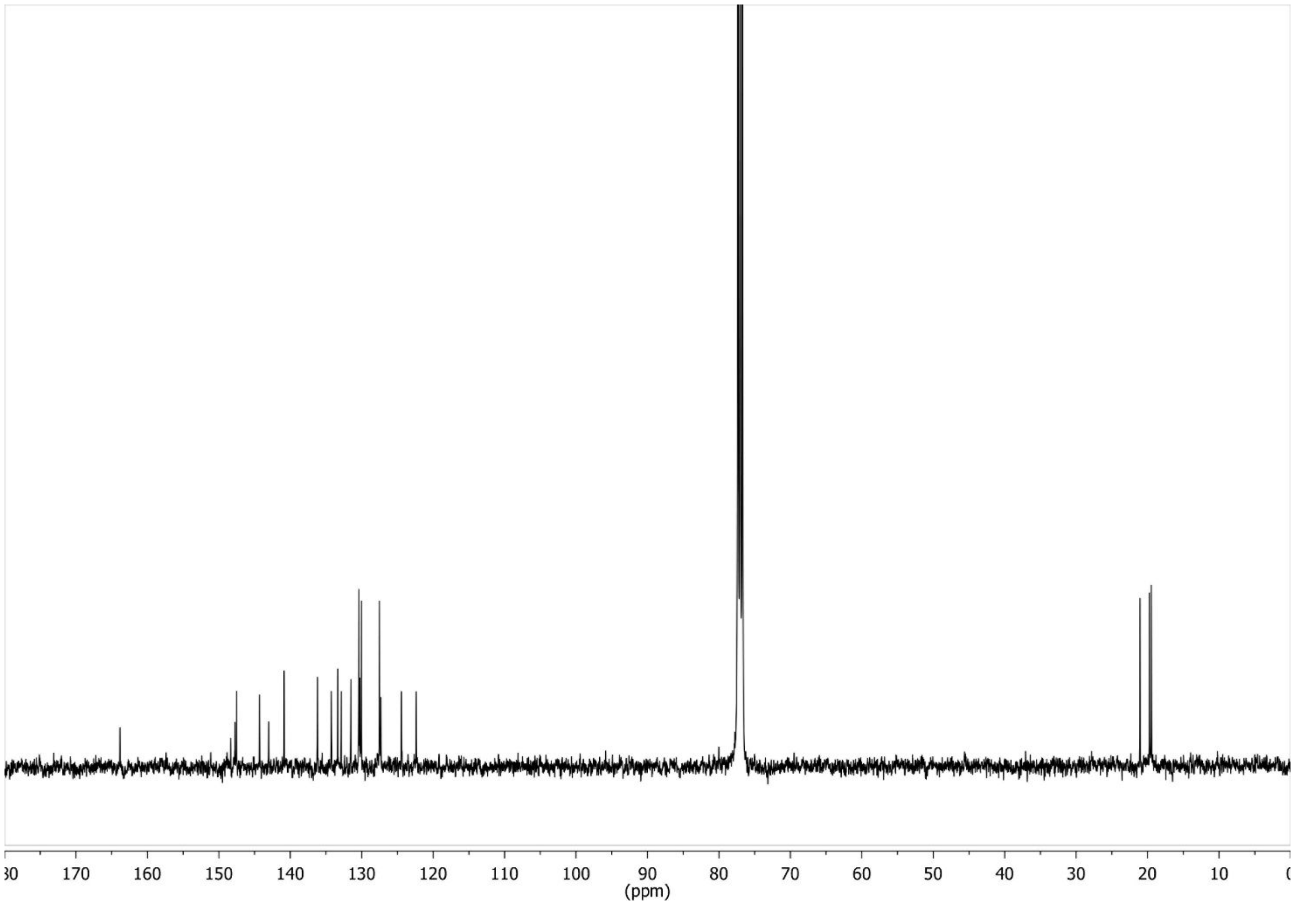

Figure S6. ^{13}C NMR spectrum (100.14 MHz, 297K, $CDCl_3$) of complex [(ppy)Au(NHC^{mes})Cl]Cl

The ^{13}C signals (Figure S6) were attributed with the help of ^{1}H-^{13}C HSQC experiment (Figure S7) at the beginning and then with the ^{1}H-^{13}C HMBC (Figure S8) to assign the resonance of the quaternary carbons and to confirm the pattern of the proton-carbon sequence. Notably frequenciesa are: C11 at 143.03 ppm (the carbon of phenylpyridine which is bonded to the gold atom); C12 at 148.34 ppm (NHC carbon bonded to Au).

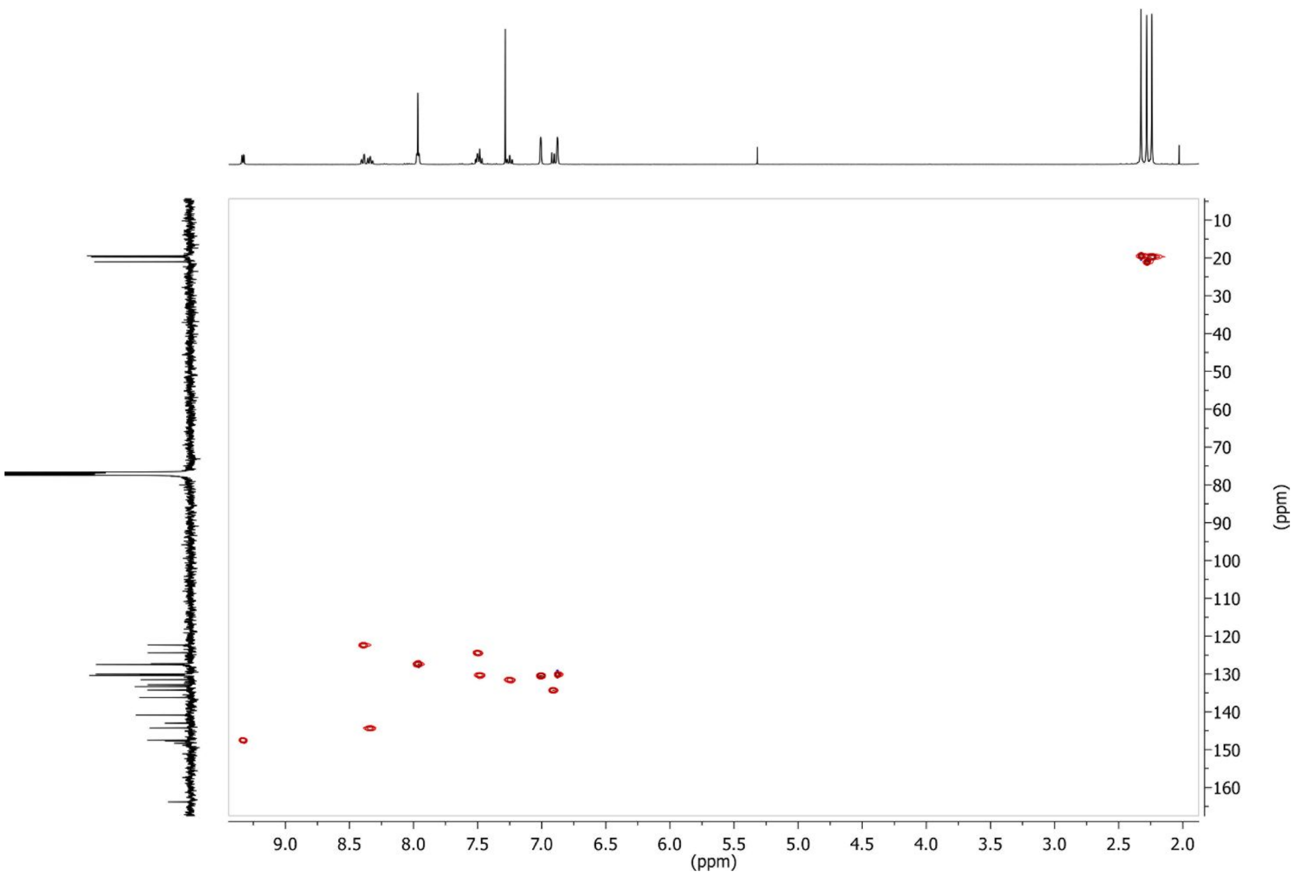

Figure S7. ^{1}H-^{13}C HSQC NMR spectrum (376.65 MHz, 297K, $CDCl_3$) of complex [(ppy)Au(NHC^{mes})Cl]Cl

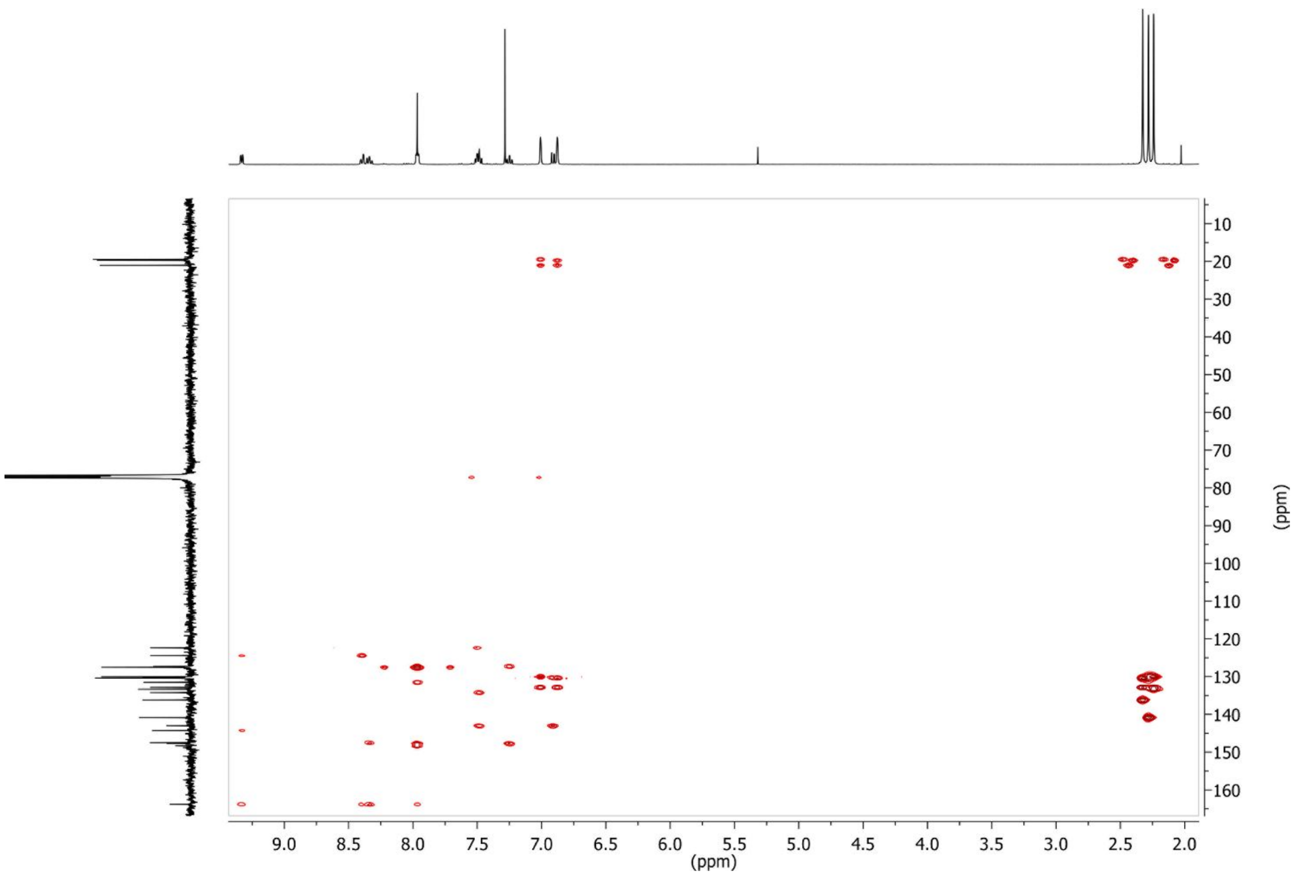

Figure S8. ^{1}H-^{13}C HMBC NMR spectrum (376.65 MHz, 297K, $CDCl_3$) of complex [(ppy)Au(NHC^{mes})Cl]Cl

^{1}H-^{15}N HMBC (Figure S9) confirm the presence of 2 types of nitrogen: one at -147.37 ppm which correlate with the proton H1 (ppy) and the other at -187.87 ppm more intense that correlate with the H13 protons (NHC^{mes}).

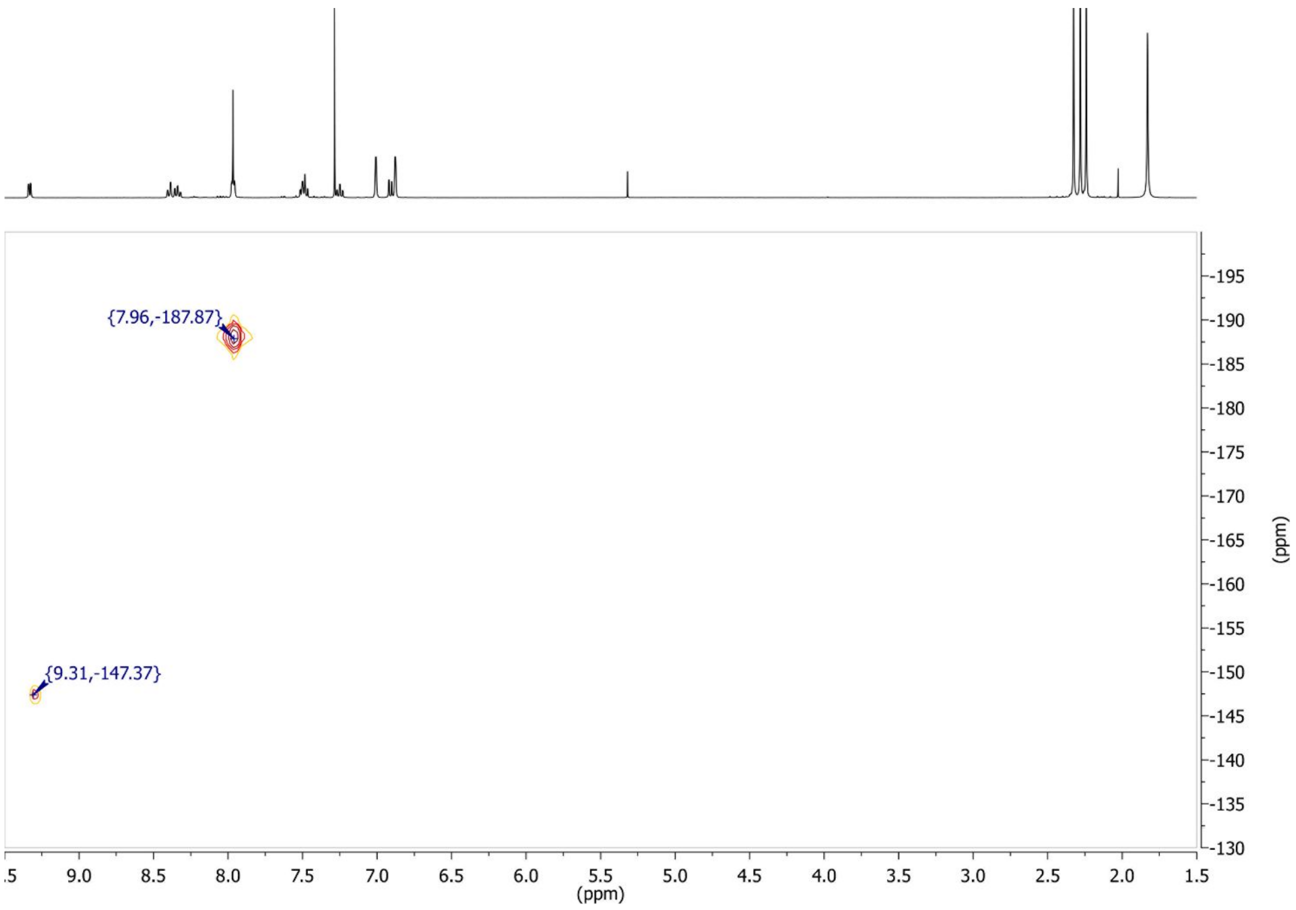

Figure S9. ^{1}H-^{15}N HMBC NMR spectrum (376.65 MHz, 297K, $CDCl_3$) of complex [(ppy)Au(NHC^{mes})Cl]Cl

Synthesis and intramolecular characterization of [(ppy)Au(NHC^{mes})OTf]OTf

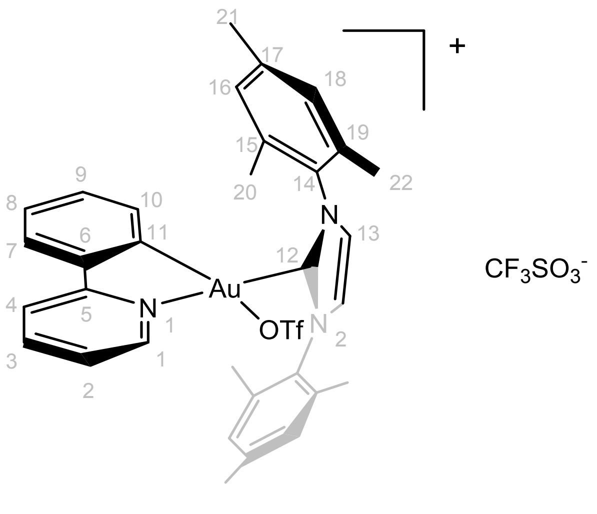

The integration in the ^{1}H NMR spectra (Figure S10) confirms the number of protons expected. It is immediately noticeable the shift of the protons of the aromatic area especially the one close to the nitrogen of the phenylpyridine (H1) which move from 9.33 (red line in the zoomed window) to 8.72 ppm (Table S1). H10 decrease at 6.75 ppm ($\Delta = 0.16$). The imidazole protons H13 present a decrease of 0.34 ppm with a chemical shift of 7.62.

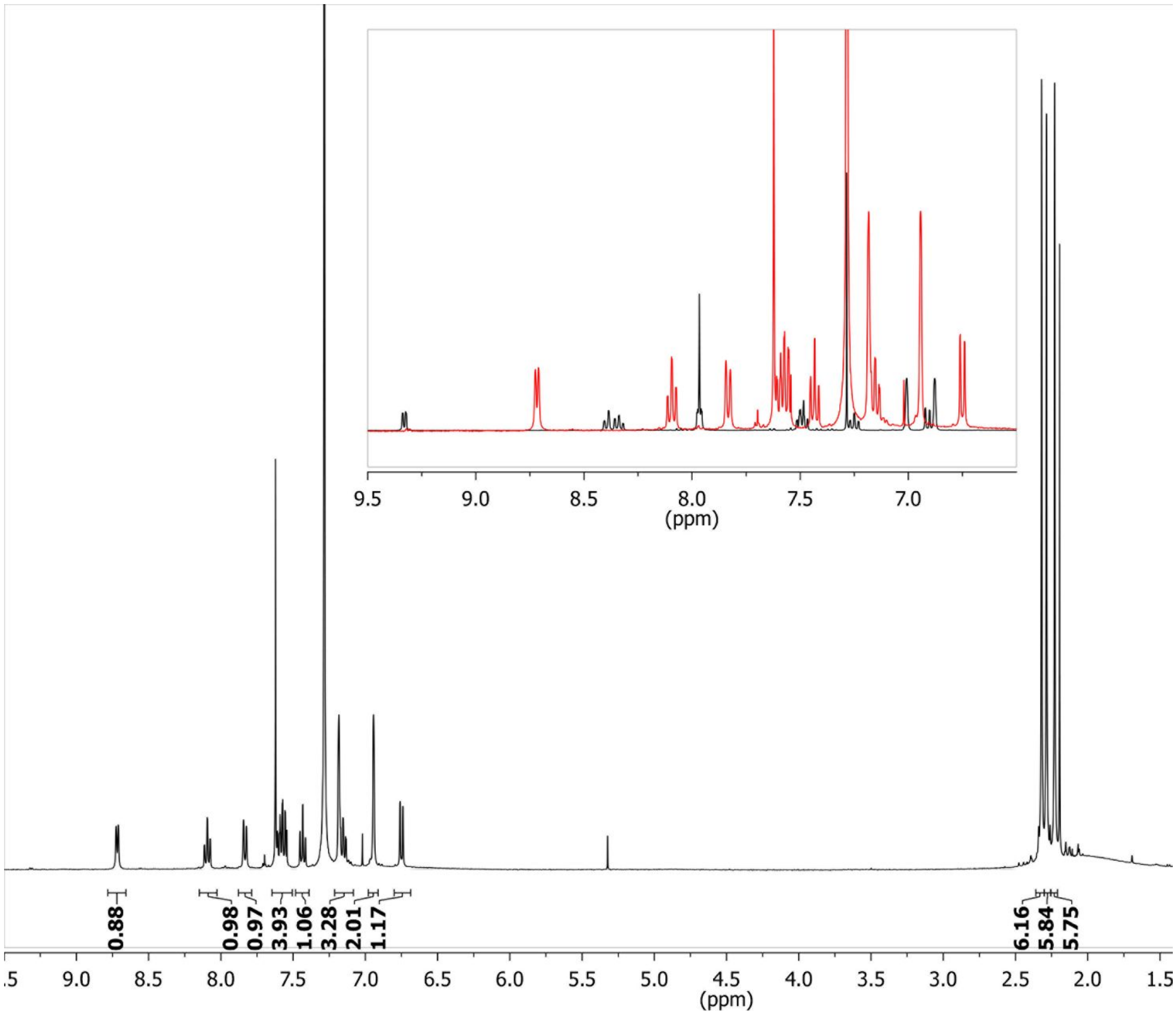

Figure S10. ^{1}H NMR spectrum (376.65 MHz, 297K, $CDCl_3$) of complex [(ppy)Au(NHC^{mes})OTf]OTf. In red a section of ^{1}H NMR spectrum (376.65 MHz, 297K, $CDCl_3$) of complex [(ppy)Au(NHC^{mes})Cl]Cl

The bi-dimensional experiment ^{1}H-^{1}H COSY (Figure S11) allowed to determine the other protons: H4 changes from 8.40 to 7.83 ppm (Δ = 0.57 ppm), H3 from 8.34 to 8.09 (Δ = 0.25 ppm), H21 at 2.32 ppm is coupled with H16 and H18 (Figure S9)

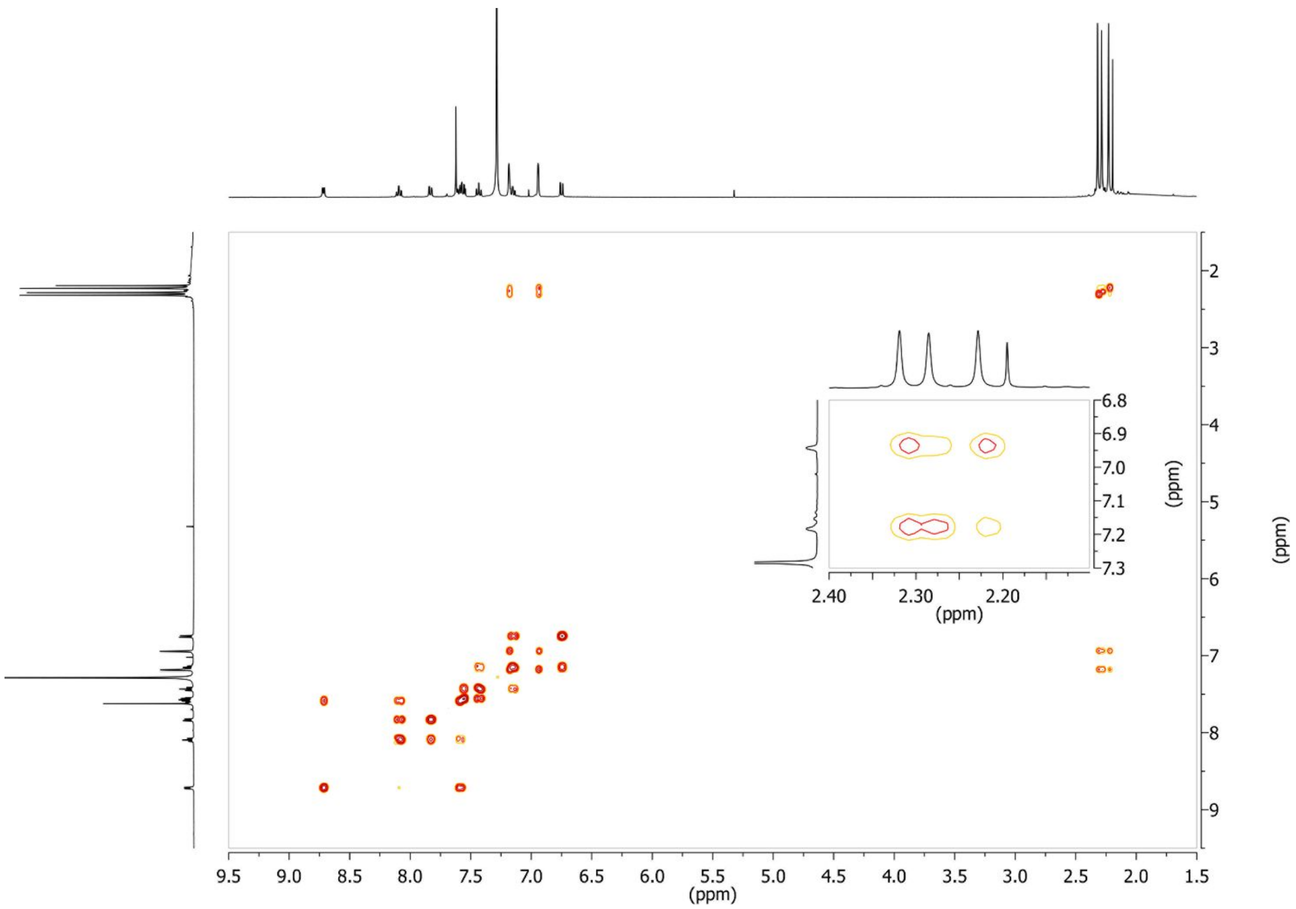

Figure S11. ^1H-^1H COSY NMR spectrum (376.65 MHz, 297K, $CDCl_3$) of complex [(ppy)Au(NHC^{mes})OTf]OTf.

The remaining signals were attributed with the help of ^1H-^1H NOESY experiment (Figures S12 and S13). The signal at 2.23 ppm gives an Overhouse Effect with the protons at 9.91 labelled as H10. This singlet, therefore, is the methyl group number as H20. The third singlet at 2.29 ppm must be the H22. The H16 and H18 are the singlet which integrate 2 protons corresponding at 6.94 and 7.18 ppm, respectively.

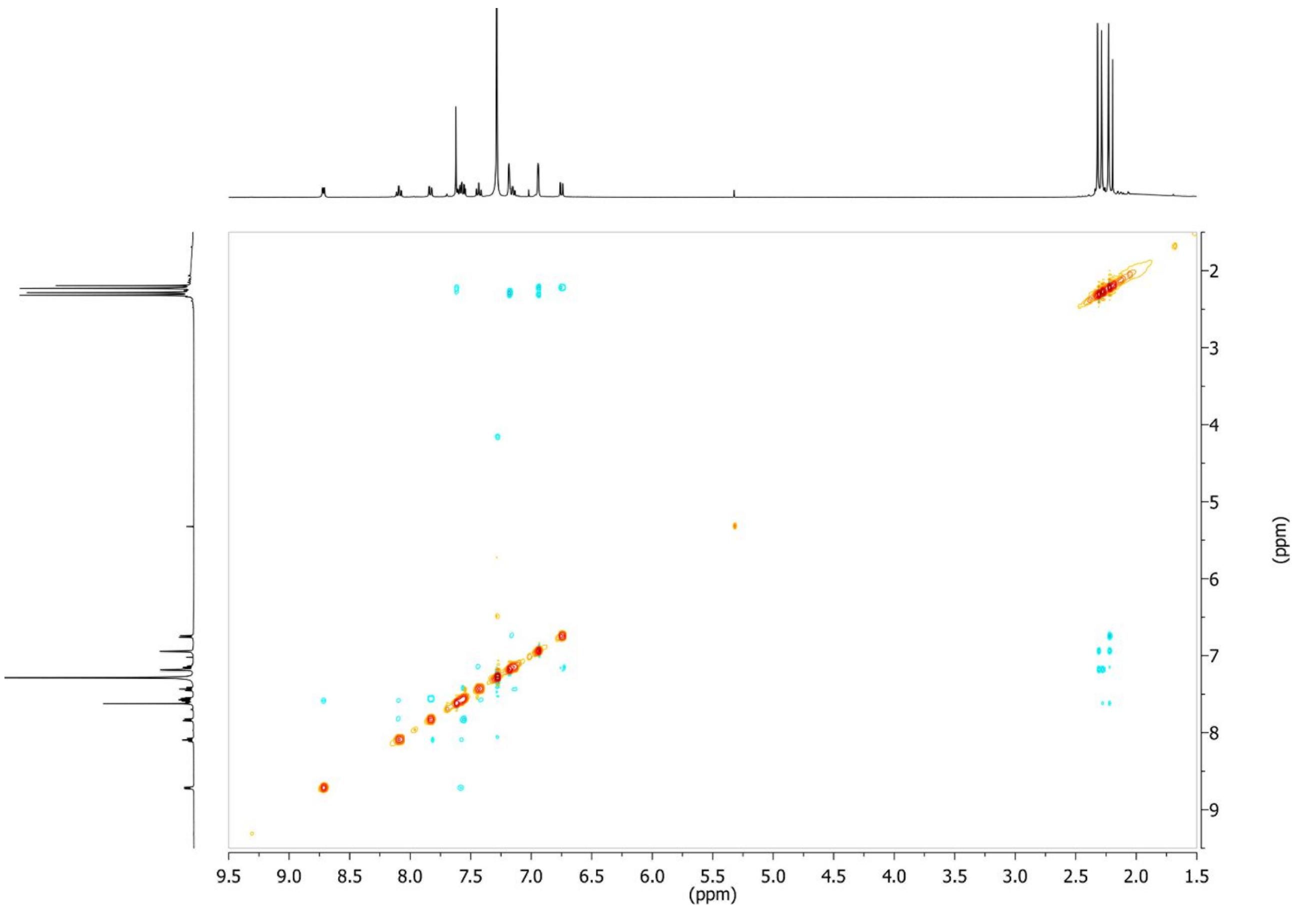

Figure S12. ^1H-^1H NOESY NMR spectrum (376.65 MHz, 297K, $CDCl_3$) of complex [(ppy)Au(NHC^{mes})OTf]OTf.

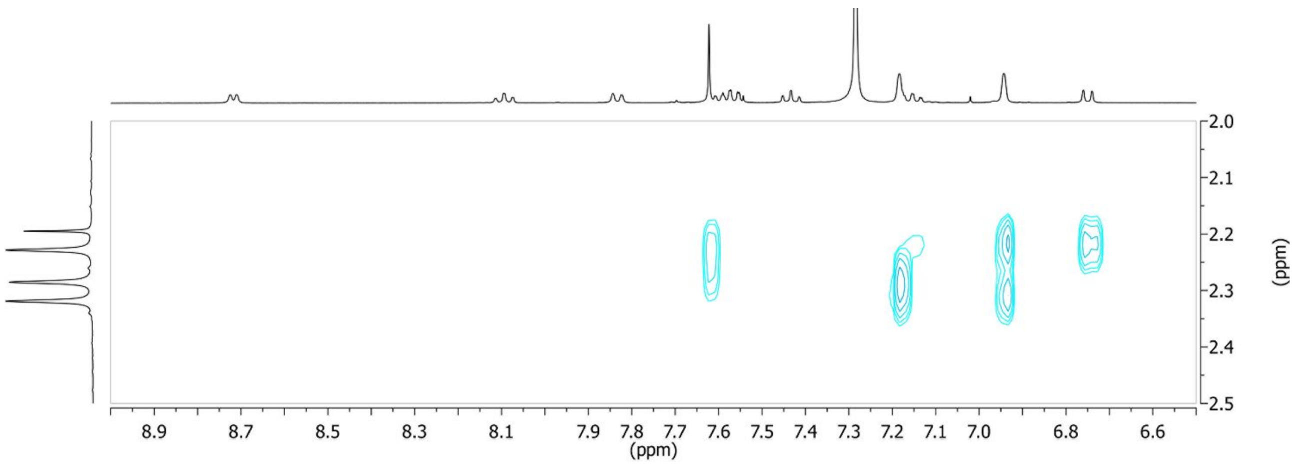

Figure S13. A section of ^1H-^1H NOESY NMR spectrum (376.65 MHz, 297K, $CDCl_3$) of complex [(ppy)Au(NHC^{mes})OTf]OTf.

The carbon chemical shifts (Figure S14) were assigned with the help of the ^{1}H-^{13}C HSQC (Figure S15) and ^{1}H-^{13}C HMBC (Figure S16) experiments and the results compared with [(ppy)Au(NHC^{mes})Cl]Cl (red line). The most significant change happened to the phenylpyridine fragment. Quaternaries carbons C5 and C6 have respectively 3.61 and 10.95 ppm chemical shift, whyle C1 increase of 2 ppm. The NHC carbons showed differences equal or less than 1 ppm. The gold-bonded carbons C11 and C12 does not present significant shifts.

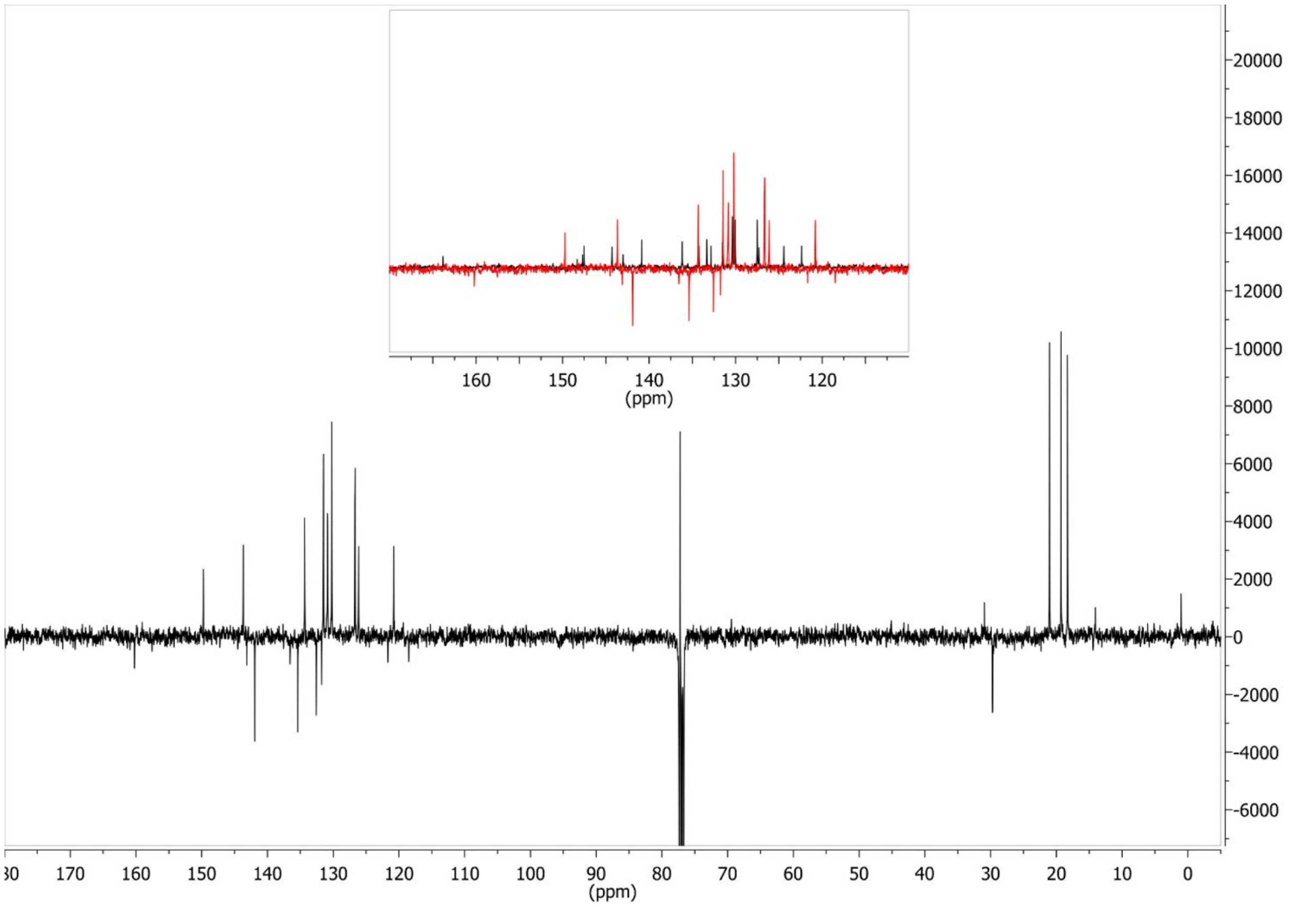

Figure S14. ^{13}C NMR spectrum (100.14 MHz, 297K, $CDCl_3$) of complex [(ppy)Au(NHC^{mes})OTf]OTf. In red a section of ^{13}C NMR spectrum (376.65 MHz, 297K, $CDCl_3$) of complex [(ppy)Au(NHC^{mes})Cl]Cl

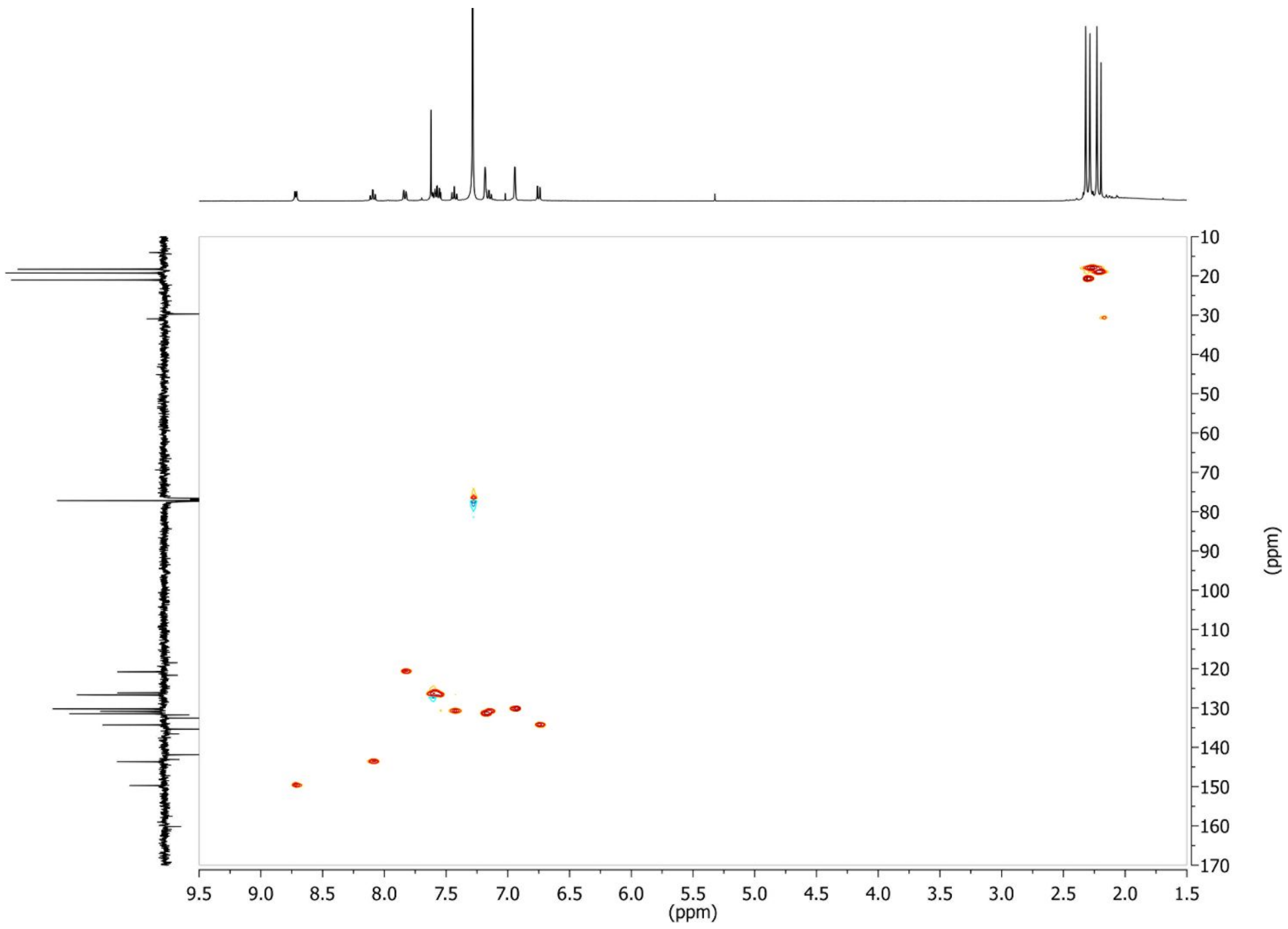

Figure S15. ^{1}H-^{13}C HSQC NMR spectrum (100.14 MHz, 297K, $CDCl_3$) of complex [(ppy)Au(NHC^{mes})OTf]OTf

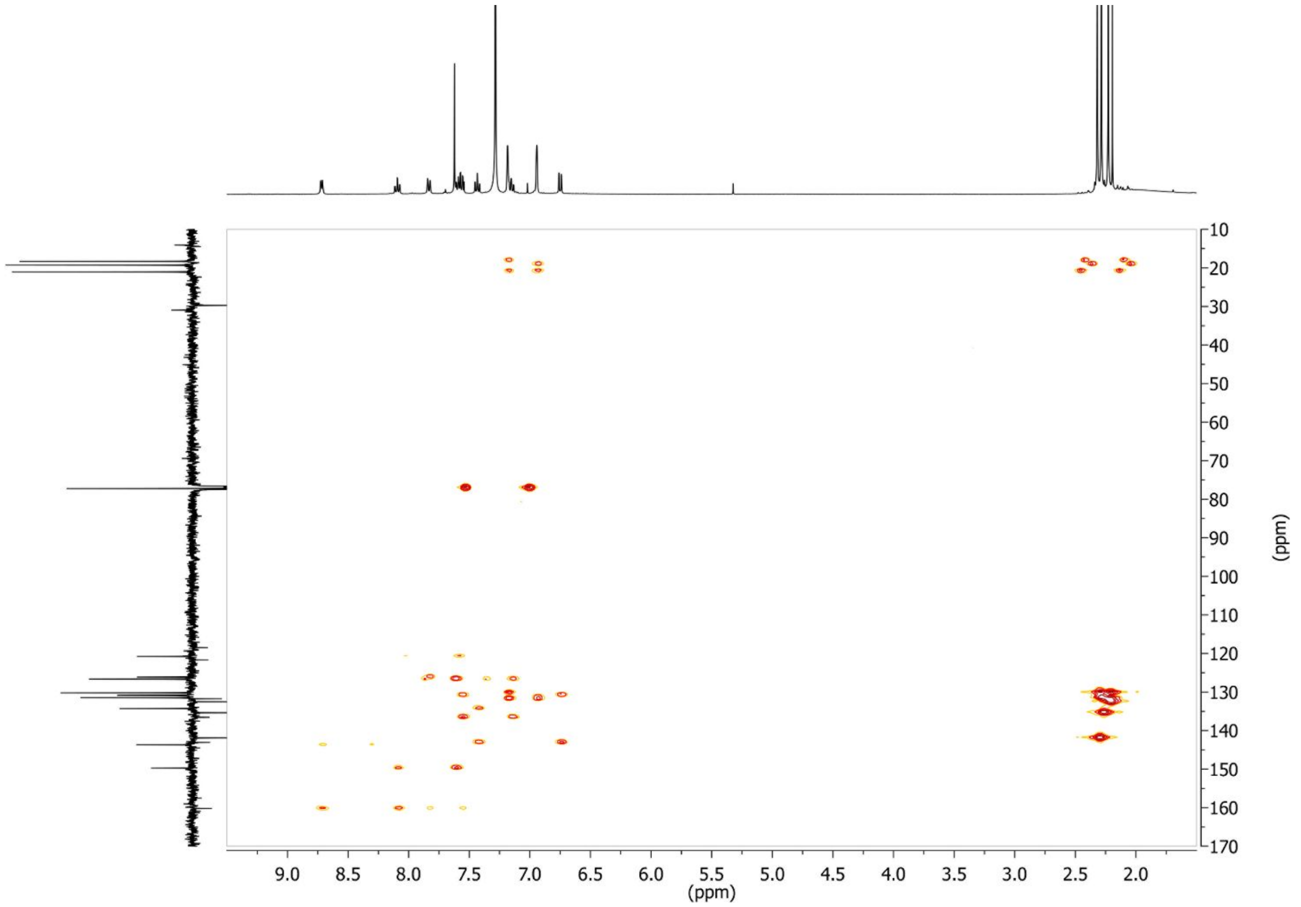

Figure S16. ^{1}H-^{13}C HMBC NMR spectrum (100.14 MHz, 297K, $CDCl_3$) of complex [(ppy)Au(NHC^{mes})OTf]OTf

^{1}H-^{15}N HMBC (Figure S17) presents the intense signal of the imidazole nitrogens at -186.7 ppm (+1.2 respect to dichloride) and 2 signals at -142.7 ppm (N1 of ppy at +4.7 respect to [(ppy)Au(NHC^{mes})Cl]Cl) corresponding to the correlation with H1 and H4.

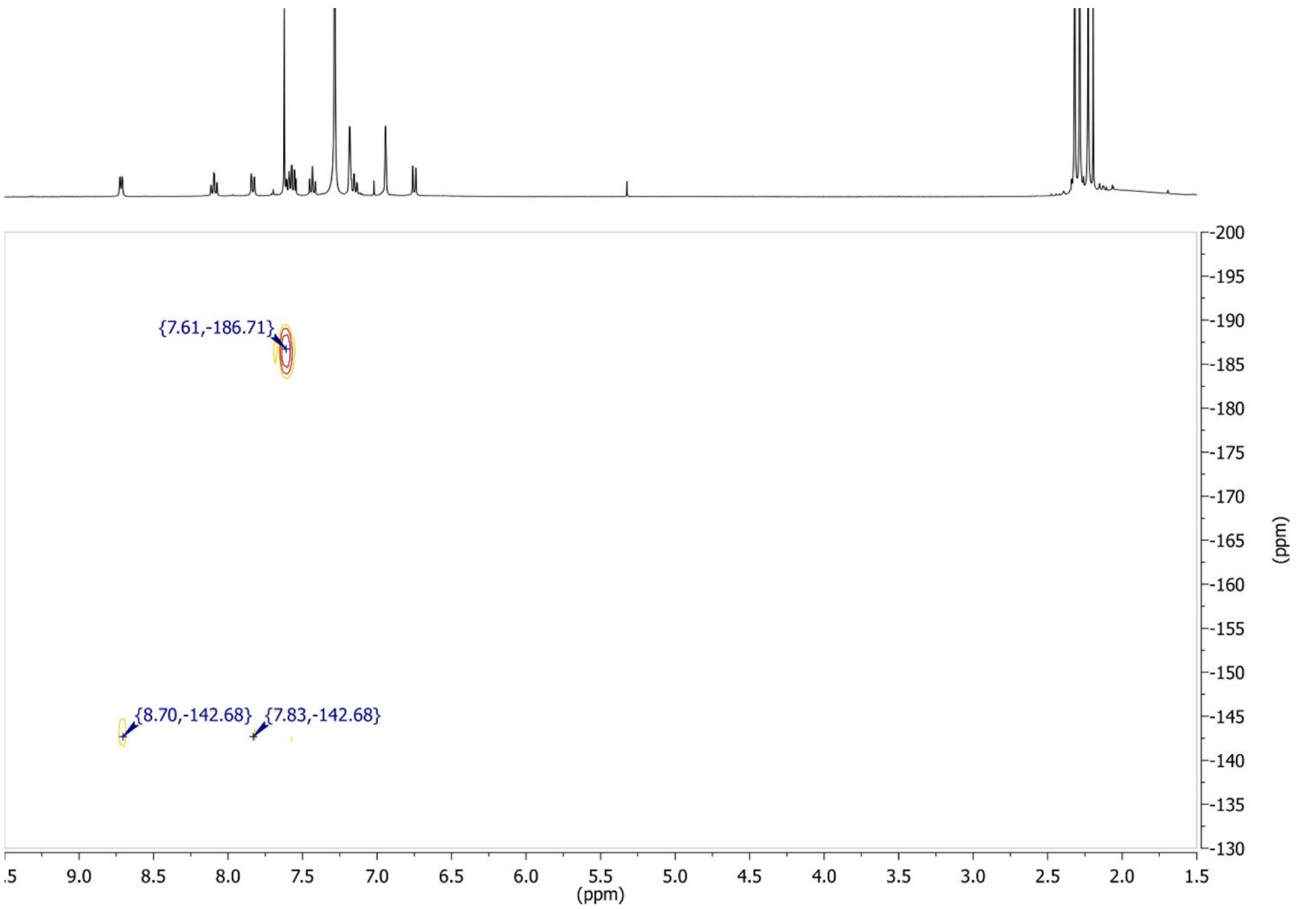

Figure S17. ^{1}H-^{15}N HMBC NMR spectrum (100.14 MHz, 297K, $CDCl_3$) of complex [(ppy)Au(NHC^{mes})OTf]OTf

Table S1

#atom	[(ppy) Au NHC^{mes} Cl]Cl (ppm)			[(ppy) Au NHC^{mes} OTf]OTf (ppm)				D (ppm)		
	^{1}H	^{13}C	^{15}N	^{1}H	^{13}C	^{15}N	^{19}F	^{1}H	^{13}C	^{15}N
1	9.33	147.72	-147.37	8.72	149.74	-142.68	-78.1	0.61	2.02	4.69
2	7.56-7.44	124.44	-187.87	7.61-7.51	126.14	-186.71		-	1.7	1.16
3	8.34	144.3		8.09	143.68			0.25	0.62	
4	8.4	122.38		7.83	120.8			0.57	1.58	
5		163.84			160.23				3.61	
6		147.52			136.57				10.95	
7	7.96	127.31		7.61-7.51	126.66			-	0.65	
8	7.56-7.44	130.27		7.43	130.88			-	0.61	
9	7.25	131.53		7.21-7.08	130.88			-	0.65	
10	6.91	134.26		6.75	134.33			0.16	0.07	
11		143.03			143.11				0.08	
12		148.34			147.21				1.13	
13	7.96	127.52		7.62	126.66			0.34	0.86	
14		132.87			131.77				1.1	
15		133.35			132.57				0.78	
16	6.86	130.05		6.94	130.23			0.08	0.18	
17		140.86			141.45				0.59	
18	7.01	130.4		7.18	131.47			0.17	1.07	
19		136.19			135.39				0.8	
20	2.24	19.74		2.23	19.29			0.01	0.45	
21	2.28	21.06		2.32	21.04			0.04	0.02	
22	2.33	19.49		2.29	18.31			0.04	1.18	

DFT calculations

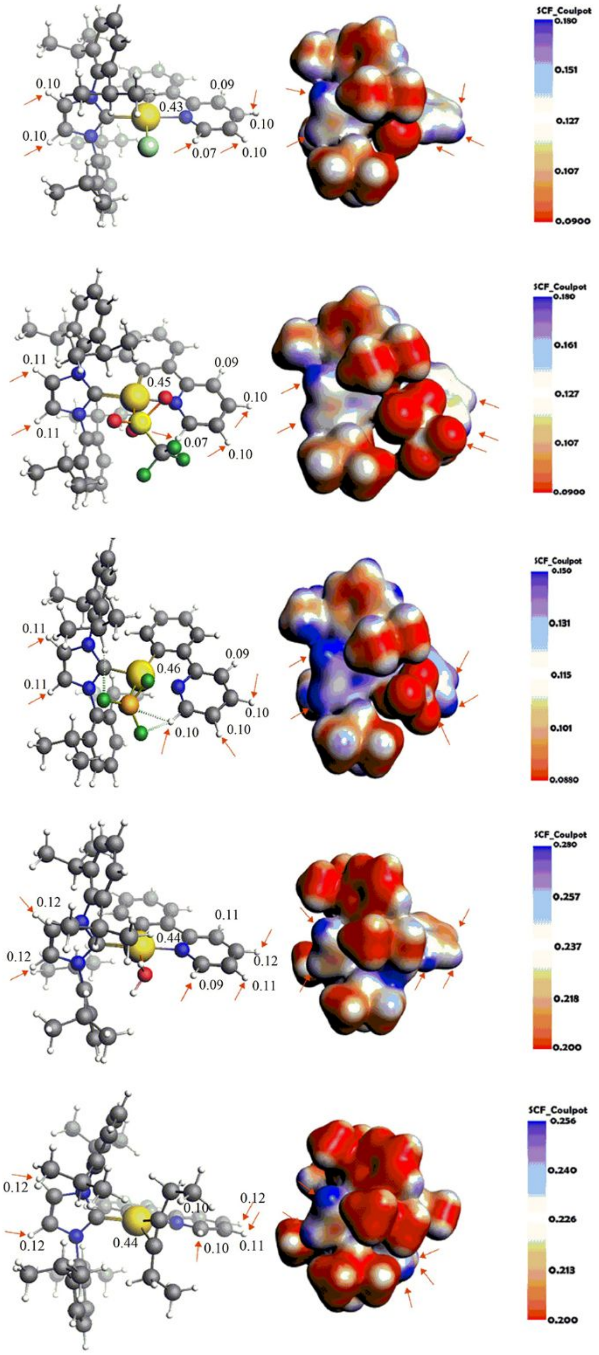

Figure S18. Optimized structures (BP86 gas phase) of [(ppy)Au(NHC^{iPr})X]$^+$ (X = Cl$^-$, BF$_4^-$, OTf$^-$) and [(ppy)Au(NHC^{iPr})Y]$^{2+}$ (Y = H$_2$O and 3-hexyne) complexes (from top to bottom: X = Cl$^-$, OTf$^-$, BF$_4^-$; Y = H$_2$O, 3-hexyne) and corresponding orientation views of the Coulomb potential mapped on an electronic isodensity surface (ρ = 0.007 e/Å^3. Coulomb potential in au). Values of VDD (e) are also shown for the most attractive hydrogen atoms and for gold.

Figure S19.

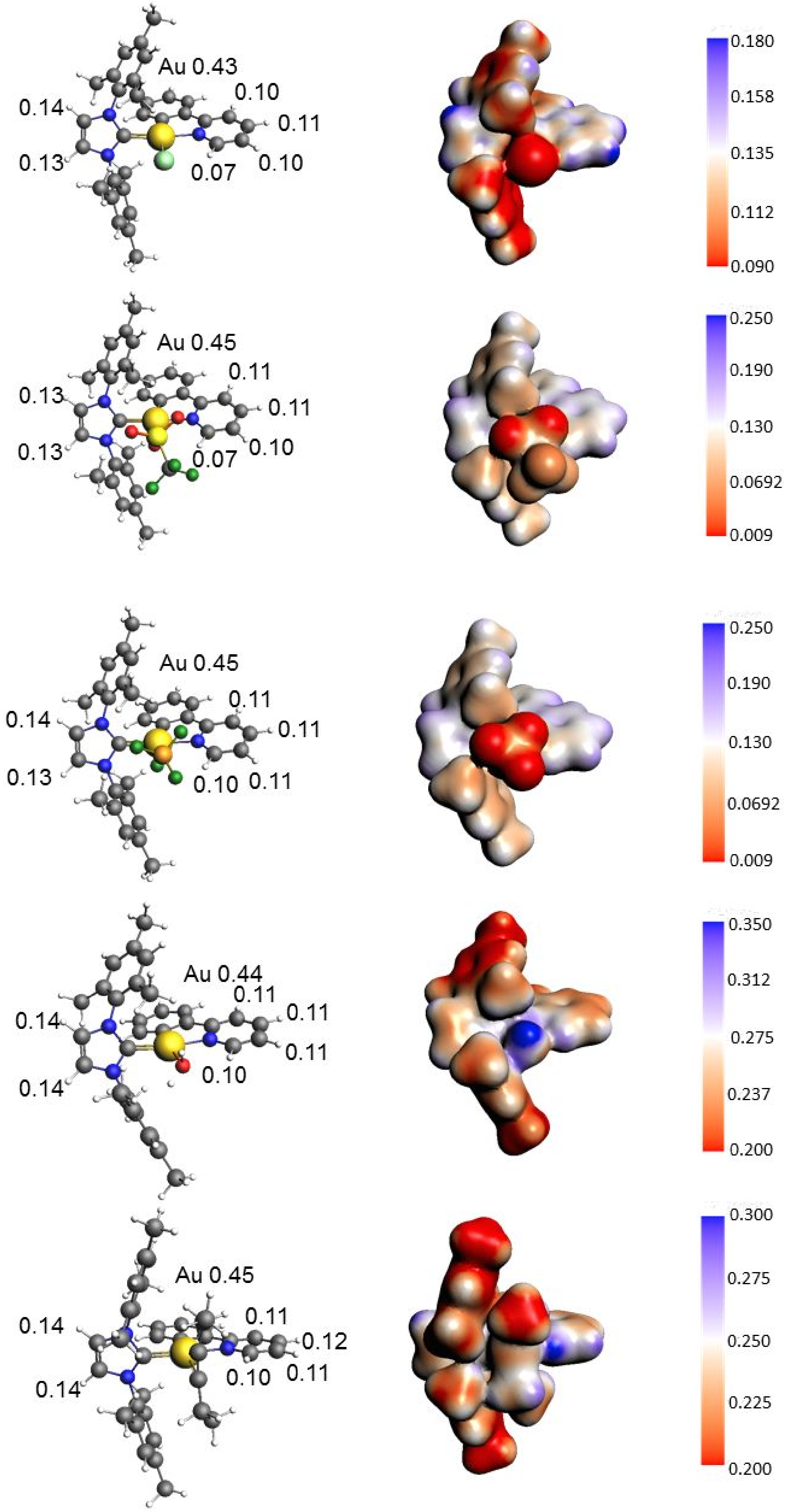
Au 0.43
0.14
0.13
0.10
0.11
0.07
0.10
0.180
0.158
0.135
0.112
0.090
Au 0.45
0.13
0.13
0.11
0.11
0.07
0.10
0.250
0.190
0.130
0.0692
0.009
Au 0.45
0.14
0.13
0.11
0.11
0.10
0.11
0.250
0.190
0.130
0.0692
0.009
Au 0.44
0.14
0.14
0.11
0.11
0.11
0.10
0.350
0.312
0.275
0.237
0.200
Au 0.45
0.14
0.14
0.11
0.12
0.10
0.11
0.300
0.275
0.250
0.225
0.200

Optimized structures (BP86-D3BJ1.0 COSMO) of [(ppy)Au(NHC^{mes})X]$^+$ (X = Cl^-, BF_4^-, OTf^-) and [(ppy)Au(NHC^{mes})Y]$^{2+}$ (Y = H_2O and 3-hexyne) complexes (from top to bottom: X = Cl^-, OTf^-, BF_4^-; Y = H_2O, 3-hexyne) and corresponding orientation views of the Coulomb potential mapped on an electronic isodensity surface (ρ = 0.007 e/Å^3. Coulomb potential in au). Values of VDD (e) are also shown for the most attractive hydrogen atoms and for gold.

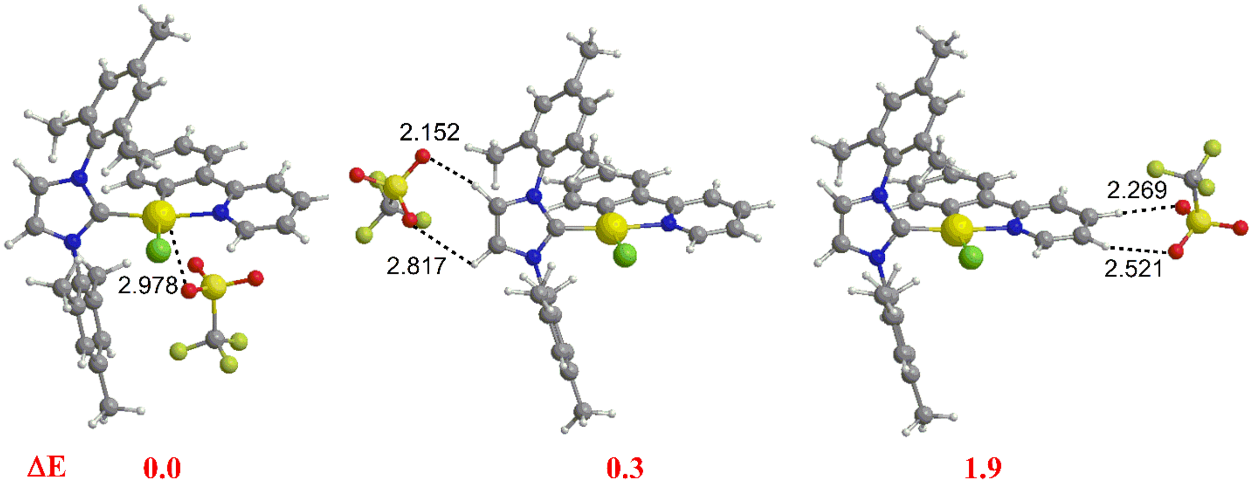

Figure S20. Optimized structures of [(ppy)Au(NHC^{mes})Cl]OTf ion pairs: anion close to gold (OTf---Au, orange region of Figure 1, Au side) (left), to H13 acidic hydrogen atoms of NHC^{mes} (OTf---H13, red region of Figure 1, NHC side) (middle) and to ppy acidic hydrogens H2 and H3 (OTf---H2/H3, blue region of Figure 1, ppy-N side) (right). Relevant distances (Å) and relative energies (ΔE in kcal/mol) of ion pairs are also shown (see Computational Details).

Evaluation of the dispersion correction and solvent effects on the relative stability of the ion pair conformations

In Table S2, the relative energies ΔE (kcal/mol) of [(ppy)Au(NHC^{iPr})Cl]OTf ion pairs in the three considered conformations (see Figure S21) calculated at different levels of theory are compared. Results are obtained by the using different computational protocols: BP86(g): geometry optimization in the gas phase without including dispersion correction and solvation; BP86-D3BJ(g): Grimme 3 BJDAMP dispersion correction included by single point calculations on optimized BP86 gas phase structures (S6 = 1.0); BP86-D3BJ solv(g): solvation included by the COSMO model (dichloromethane as solvent) by single point BP86-D3BJ calculations on optimized BP86 gas phase structures.

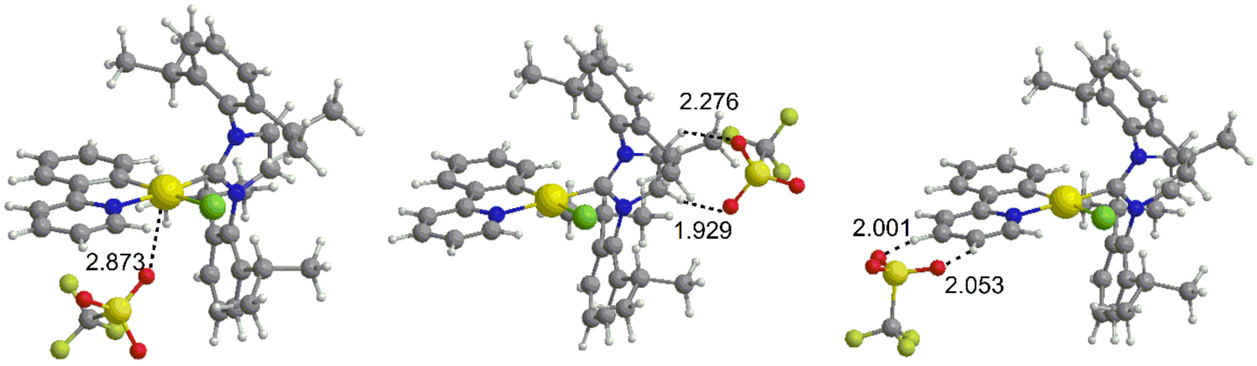

Figure S21. Optimized structures at BP86 level in the gas phase of [(ppy)Au(NHC^{iPr})Cl]OTf ion pairs: anion close to gold (OTf---Au) (left), to H13 acidic hydrogen atoms of NHC^{iPr} (OTf---H13) (middle) and to ppy acidic hydrogens H2 and H3 (OTf---H2/H3) (right). Relevant distances (Å) are also shown.

Table S2. Relative energies (ΔE in kcal/mol) of [(ppy)Au(NHC^{iPr})Cl]OTf ion pairs: anion close to gold (OTf---Au), to H13 acidic hydrogen atoms of NHC^{iPr} (OTf---H13) and to ppy acidic hydrogens H2 and H3 (OTf---H2/H3) (Figure S21) calculated at different levels of theory.

	ΔE OTf---Au	ΔE OTf---H13	ΔE OTf---H2/H3
BP86(g)	3.4	0.0	6.8
BP86-D3BJ(g)	0.0	4.9	14.1
BP86-D3BJ solv(g)	0.0	1.3	4.2

The most stable configuration for the [(ppy)Au(NHC^{iPr})Cl]OTf ion pairs in gas phase at BP86-D3BJ(g) level of theory, with the dispersion correction included in the calculations, is that where OTf$^-$ is positioned close to the metal center (OTf---Au) (Figure S21, left). Structures where the

counterion is located at the NHC^{iPr} (OTf---H13) (Figure S21, middle) and ppy (OTf---H2/H3) (Figure S21, right) side are less stable by 4.9 and 14.1 kcal/mol, respectively. As expected, the noncovalent interactions between OTf^- (at the three different positions) and the surrounding environment influence the relative stability of the three ion pair conformations. This effect can be explicitly estimated by comparing BP86(g) and BP86-D3BJ(g) energies for the three ion pairs. When dispersion correction is not included (BP86(g)), the most stable configuration for the [(ppy)Au(NHC^{iPr})Cl]OTf ion pairs is that where OTf^- is at the NHC^{iPr} side (OTf---H13), whereas configurations where the counterion is located at the Au (OTf---Au) and at the ppy (OTf---H2/H3) side are less stable by 3.4 and 6.8 kcal/mol, respectively. This result shows that the OTf^- position close to Au is mainly stabilized by van der Waals interactions and a one-to-one mapping between the Coulomb potential (Figure S18) and the anion electrostatic interaction cannot be found. In addition, one may expect that noncovalent interactions are affected by the presence of the solvent. The solvent (dichloromethane) effect can be evaluated from inspection of the values in the third row of Table S2 (BP86-D3BJ solv(g)). Indeed, solvation significantly relieves the stabilizing van der Waals contribution to the OTf---Au conformation to the extent that the potential energy surface is now relatively flat. i.e. the three conformations lie much closer in energy. The largest solvent effect on the OTf---Au configuration nicely agrees with its value of the dipole moment being the lowest in the series (14.4 D vs. 26.4 D for OTf---H13 and 34.4 D for OTf---H2/H3). As expected, the solvent also affects the geometries by increasing the OTf---Au, OTf---H13 and OTf---H2/H3 distances (compare Figure S21 and Figure 6 in the main text).

In Table S3, the relative energies ΔE (kcal/mol) of [(ppy)Au(NHC^{iPr})Cl]OTf, [(ppy)Au(NHC^{mes})OTf]OTf and [(ppy)Au(NHC^{mes})Cl]OTf ion pairs in the three considered conformations (see Figures 6 and 7 in the main text and Figure S20) calculated using optimized structure at BBP86-D3BJ COSMO (dichloromethane) (i.e. including both dispersion and solvent effects) level of theory are compared. Results are obtained by using different methods for the dispersion correction evaluation: Grimme 3 BJDAMP (D3BJ1.0 with S6 = 1.0, default value in ADF

code), the Grimme 3 BJDAMP (D3BJ0.64 with S6 = 0.64, parametrization value recommended in J. Phys. Chem. Lett. 2016, 7, 2197) and the Grimme 3 (D3) dispersion corrections, which are included by single point calculations on optimized BBP86-D3BJ COSMO solvent phase structures. From Table S3, a very flat potential energy surface emerges, with the three different configurations very close in energy at the three levels of theory and for all the [(ppy)Au(NHC^{iPr})Cl]OTf, [(ppy)Au(NHC^{mes})OTf]OTf and [(ppy)Au(NHC^{mes})Cl]OTf ion pairs. Notably, when dispersion correction is included at D3BJ1.0 and D3 level, the most stable configuration for the [(ppy)Au(NHC^{iPr})Cl]OTf ion pairs is that where OTf^{-} is located at the Au (OTf---Au) side, with configurations at the NHC^{iPr} (OTf---H13) and ppy (OTf---H2/H3) side being only slightly less stable by 0.3-0.6 kcal/mol and 3.2-3.7 kcal/mol, respectively. A different trend is calculated at D3BJ0.64 level. In this case the most stable configuration is that where OTf^{-} is located at the NHC^{iPr} (OTf---H13) side, consistent with the experimental data, with configurations at the Au (OTf---Au) and ppy (OTf---H2/H3) side being less stable by 1.0 and 2.6 kcal/mol, respectively.

Table S3. Relative energies (ΔE in kcal/mol) of [(ppy)Au(NHC^{iPr})Cl]OTf, [(ppy)Au(NHC^{mes})Cl]OTf and [(ppy)Au(NHC^{mes})OTf]OTf ion pairs: anion close to gold (OTf---Au), to H13 acidic hydrogen atoms of NHC^{iPr} (NHC^{mes}) (OTf---H13) and to ppy acidic hydrogens H2 and H3 (OTf---H2/H3) (Figures 6 and 7 in the main text and Figure S20, respectively) calculated at different levels of theory for dispersion correction inclusion.

[(ppy)Au(NHC^{iPr})Cl]OTf	ΔE OTf---Au	ΔE OTf---H13	ΔE OTf---H2/H3
D3BJ1.0	0.0	0.3	3.2
D3BJ0.64	1.0	0.0	2.6
D3	0.0	0.6	3.7

[(ppy)Au(NHC^{mes})OTf]OTf	ΔE OTf---Au	ΔE OTf---H13	ΔE OTf---H2/H3
D3BJ1.0	0.0	3.2	5.1
D3BJ0.64	0.0	1.9	3.6
D3	0.0	4.5	6.2

[(ppy)Au(NHC^{mes})Cl]OTf	ΔE OTf---Au	ΔE OTf---H13	ΔE OTf---H2/H3
D3BJ1.0	0.0	1.5	3.6
D3BJ0.64	0.0	0.3	1.9
D3	0.0	2.1	4.1

Evaluation of the coordination ability of $[(ppy)Au(NHC^{iPr})]^{2+}$ towards Cl^-, OTf^- and BF_4^-

Table S4. X (X = Cl^-, OTf^-, BF_4^-) bonding electronic energies ΔE and Gibbs free energies ΔG to $[(ppy)Au(NHC^{iPr})]^{2+}$. Values are in kcal/mol.

	Cl^-	OTf^-	BF_4^-
ΔE	-54.3	-34.3	-24.7
ΔG	-44.0	-17.5	-12.5

[1] Sabatelli F.; Segato J.; Belpassi, L.; Del Zotto, A.; Zuccaccia, D.; Belanzoni, P. 'Monitoring of the Pre-Equilibrium Step in the Alkyne Hydration Reaction Catalyzed by Au(III) Complexes: A Computational Study Based on Experimental Evidences.' *Molecules*, **2021**, *26*, 2445.
